# Controlling Photoconductivity in PBI Films by Supramolecular Assembly

**DOI:** 10.1002/chem.201800201

**Published:** 2018-02-19

**Authors:** Emily R. Draper, Lewis J. Archibald, Michael C. Nolan, Ralf Schweins, Martijn A. Zwijnenburg, Stephen Sproules, Dave J. Adams

**Affiliations:** ^1^ School of Chemistry University of Glasgow Glasgow G12 8QQ UK; ^2^ Department of Chemistry University of Liverpool Liverpool L69 7ZD UK; ^3^ Institut Laue-Langevin Large Structures Group 71 Avenue des Martyrs, CS 20156 38042 Grenoble CEDEX 9 France; ^4^ Department of Chemistry University College London 20 Gordon Street London WC1H 0AJ UK

**Keywords:** aggregation, perylene, perylene bisimide, photoconductivity, self-assembly

## Abstract

Perylene bisimides (PBIs) self‐assemble in solution. The solubility of the PBIs is commonly changed through the choice of substituents at the imide positions. It is generally assumed this substitution does not affect the electronic properties of the PBI, and that the properties of the self‐assembled aggregate are essentially that of the isolated molecule. However, substituents do affect the self‐assembly, resulting in potentially different packing in the formed aggregates. Here, we show that the photoconductivity of films formed from a library of substituted PBIs varies strongly with the substituent and demonstrate that this is due to the different ways in which they pack. Our results open the possibility for tuning the optoelectronic properties of self‐assembled PBIs by controlling the aggregate structure through careful choice of substituent, as demonstrated by us here optimising the photoconductivity of PBI films in this way.

Perylene bisimides (PBIs, or perylene diimides, PDIs) can self‐assemble into a range of aggregates in solution, many of which can be used to prepare electronic materials.^1^ For example, PBIs can be used in *p*‐n heterojunctions, as part of a photocatalytic system for hydrogen production and in perovskite solar cells for example. PBIs are ideal candidates for light harvesting materials due to their broad UV/Vis absorption and thermal stability.1b

PBIs are however often poorly soluble. Functionalization of a PBI at the imide position is often used to improve solubility.1a To render them soluble in organic solvents, PBIs are generally substituted with long alkyl chains, whereas solubility in aqueous media is achieved using oligo(ethylene oxide) or ionisable groups.1a, 2 Solubility of the PBIs is crucial to enable processing of the materials into devices. Based on the literature, functionalization at the imide position with such (simple) substituents generally should not affect the electronic properties of the molecules.^3^ As such, the function of the PBI is often expected to be similar irrespective of the solubilising group.

We have previously studied the self‐assembly of five PBIs functionalized at the imide position with amino acids in water at both high pH and low pH.^4^ We used these to form photoconductive thin films. The films responded most effectively to UV light. Here, we show that, in contrast to what would be expected from the literature, the optoelectronic properties of a series of such PBI films, such as their photoconductivity and the amount of free charges formed, vary strongly with the chosen amino acid substituent. The origin of this variation appears to be effect of the substituent on the structures of self‐assembled aggregate formed rather than a change in the inherent properties of the constituting molecules.

Eight different PBIs were synthesized using literature procedures,4a, 5 functionalized at the imide position using alanine (**PBI‐A**), phenylalanine (**PBI‐F**), histidine (**PBI‐H**), leucine (**PBI‐L**), serine (**PBI‐S**), valine (**PBI‐V**), tryptophan (**PBI‐W**), or tyrosine (**PBI‐Y**) (Figure 1). To the best of our knowledge, only **PBI‐S** has not been previously reported. Self‐assembly was investigated at high pH (pH 8; we add one equivalent of sodium hydroxide to each PBI, so on average a singly deprotonated material should be formed) at a concentration of 5 mg mL^−1^. Since these act as surfactants, the aggregation state is likely to be concentration dependent, so all data were collected at this same concentration.

**Figure 1:**
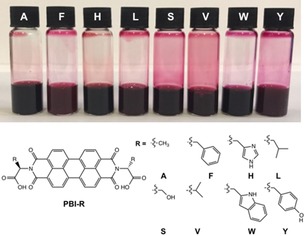
(Top) Photograph of PBIs in solution. (Bottom) Structures of the PBIs; the letter represents the amino acid used for functionalisation.

The PBIs have different degrees and types of aggregation. UV/Vis absorption spectra of the solutions showed all samples had a weak S_0_–S_2_ transition at around 385 nm and strongly absorbing S_0_–S_1_ transitions between 450–600 nm (Figure S4, Supporting Information). Such spectra are typical for aggregated PBIs.^2^, ^6^ The intensity ratio of the split S_0_–S_1_ peaks differed for the PBIs, showing that the amino acids affect the local molecular packing. There are two apparent families. The spectra for **PBI‐A**, **PBI‐H**, **PBI‐L**, **PBI‐S** and **PBI‐V** are similar to each other (example data for **PBI‐A** in Figure 2 a). The intensity at 510 nm is greater than that at 560 nm. The spectra for the other PBIs show peaks at 510 and 560 nm which have comparable intensity (example data for **PBI‐F** in Figure 2 b).

**Figure 2:**
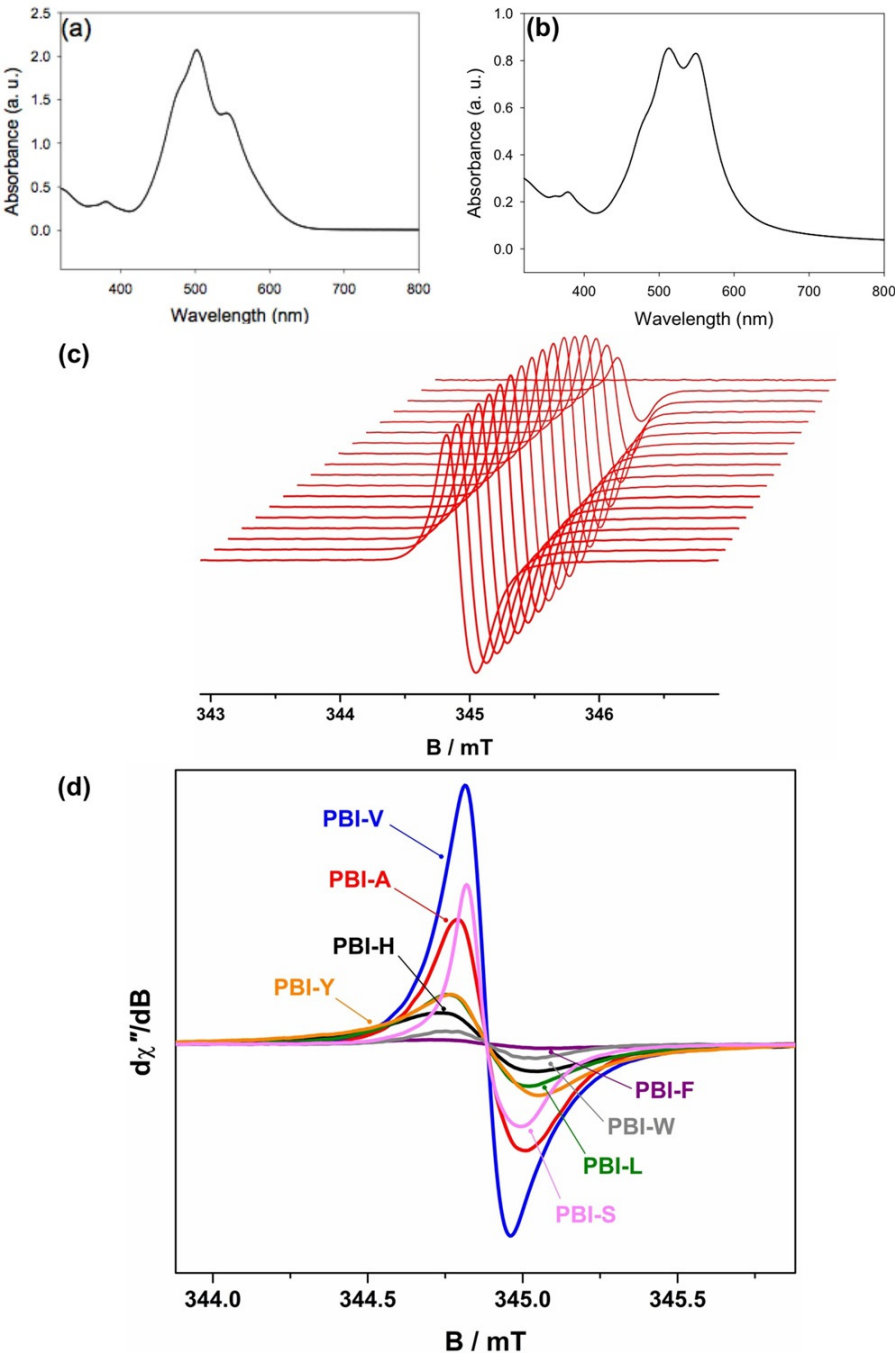
(a) UV/Vis absorption spectra of **PBI‐A** and (b) **PBI‐F** showing differences in aggregation. (c) Grow in of EPR signal in the **PBI‐A** solution after irradiation with 365 nm; spectra recorded at 2 min intervals over 33 min. (d) overlay of the normalized EPR spectra corresponding to maximum radical content for each PBI.

All of the samples showed a shear‐thinning viscosity behaviour, suggesting that there are worm‐like micelles present (Figure S1, Supporting Information).4a Small‐angle neutron scattering (SANS) data were consistent with anisotropic self‐assembled structures being present in solution (Figure S2 and S3, Tables S1–3, Supporting Information). From the fits to the SANS data, the diameters and lengths of the self‐ assembled structures vary significantly amongst the PBIs. **PBI‐F** for example forms thin fibres (radius of around 10 Å) whilst **PBI‐A** forms elliptical structures with a radius of around 50 Å, and **PBI‐S** elliptical structures with a radius of around 100 Å. There is no simple correlation between the molecular packing and the self‐assembled structure. For example, **PBI‐A** and **PBI‐S** both form elliptical structures (by SANS), but with different radii; the UV/Vis absorption spectra are however very similar.

This implies that self‐assembly might be taking place on two distinct length scales; the molecular length scale, where individual molecules stack together, and a larger scale where these primary aggregates aggregate together to form larger structures.

For these PBIs, photoconductivity arises from the formation of the radical anion.4a, 4b, 7 We have previously discussed in detail the wavelength dependence for the photoconductivity of such PBIs,4a and have shown that photoconductivity only arises when the samples are irradiated with light <400 nm, with 365 nm being optimal. This correlates with the formation of the radical anion at these wavelengths. The ability to form a radical anion upon irradiation was initially investigated by exposure of the solution in a sealed cuvette to light from a 365 nm LED. We have shown that this wavelength is necessary to excite from S_0_ to S_2_ to generate free charge carriers in dried films;4a to compare to the data collected on films (see below), we also used this wavelength for the solutions. From a thermodynamic perspective, the key parameter for radical formation and photoconductivity is the ion‐pair energy (or fundamental gap), the energy required to generate free charges. After irradiation, the UV/Vis absorption spectra for the solutions showed that the radical anion was formed by the presence of new peaks at 720, 810 and 975 nm (Figure S6, Supporting Information).^8^ The concentration of radical anion could not be calculated from the spectra due to the coincidental formation of the dianion in some cases, which absorbs at 620 nm.8b Instead, electron paramagnetic resonance (EPR) was used to quantify the radical anion species, as the dianion is EPR‐silent. TEMPO in water at the same concentration as the PBIs in solution was used as a standard to quantify the amount of radical generated. The signal generated from irradiation of the PBIs was monitored until it reached a plateau (Figure 2 c and Figures S7 and S8, Supporting Information) and the maximum value was taken. In all cases, a featureless isotropic signal with *g*≈2.0033 was observed, typical of a PBI radicals;^9^ the radical content ranged from 2.2 % for **PBI‐W** and **PBI‐F** up to 12.5 % for **PBI‐V** (Table S12).

To investigate whether functionalization at the imide position affects the ion‐pair energy (difference between ionization potential and electron affinity) and if that can explain the differences in radical content, we performed a combination of ΔDFT calculations (B3LYP+COSMO solvation model using *ϵ*_r_ 80, for more details see Supporting Information)) and cyclic voltammetry (CV) measurements.^10^ The electron affinity values calculated using ΔDFT (Tables S8–S11, Supporting Information) are similar to data for other imide‐substituted PBIs,^11^ and importantly show negligible variation in between the different PBIs. Similarly, the ionisation potential predicted by ΔDFT, as well as the ion‐pair energy are predicted to show little variation. Experimentally, the onset of light absorption, *λ*_onset_, measured by UV/Vis absorption spectra are also very similar for all aqueous solutions of PBIs and correspond to an optical gap (*E*_g_) of ≈1.8 eV (Table S4, Supporting Information). This value is very similar to the calculated ion‐pair energies, suggesting that little extra energy is required in an aqueous solution to generate PBIs radicals from PBIs with a neutral excited state. The electron affinities measured using CV in water at high pH (Table S4–7, and Figure S4–S5, Supporting Information) were found to be very close to the calculated values and importantly display the same lack of variation with amino acid. We could not explicitly measure the ionisation potentials for the PBIs in the aqueous solutions as this lies outside the stability field of water.

Hence, from the data above, along with the CV and ΔDFT results, it is clear that the PBIs form different types of aggregate in solution, but the expected fundamental electronic properties based on those of the isolated molecules are similar. The concentrations of radical anion and dianion produced on irradiation are however different. This implies that either the fundamental electronic properties of the aggregates are different from that of the isolated molecules or some other (kinetic) factor dominates. We note that aggregation has been shown elsewhere to affect the properties of PBIs.^12^

Films of the PBIs were prepared by drying the solutions in a mask between two electrodes. The current was then measured in the dark and after irradiation with a 365 nm LED. We ensured continuous films had been formed on drying with no crystallisation by viewing under a cross‐polarised light microscope (Figure S10, Supporting Information). Scanning electron microscopy (SEM) revealed that the films for the PBIs except for **PBI‐F** and **PBI‐W** contained fibre‐like structures (Figure S9, Supporting information). This agrees with the SANS data and viscosity data that worm‐like‐micelles are present in solution; these clearly persist on drying. The fibrous structures are between 10–15 nm in diameter, generally in line with the diameters for the structures in solution.

The films showed the greatest photoresponse when irradiated at 365 nm (Figure S12–S20, Supporting Information). While the ionization potential and electron affinity values are similar (see above), the photoconductivities varied significantly. **PBI‐A**, **PBI‐S**, and **PBI‐V** showed much larger responses to irradiation than the other PBIs, with the newly reported **PBI‐S** being best in class. **PBI‐L**, **PBI‐H**, and **PBI‐Y** showed a much lower response, and **PBI‐F** and **PBI‐W** showed very little response to the light. (Figure 3 a).

**Figure 3:**
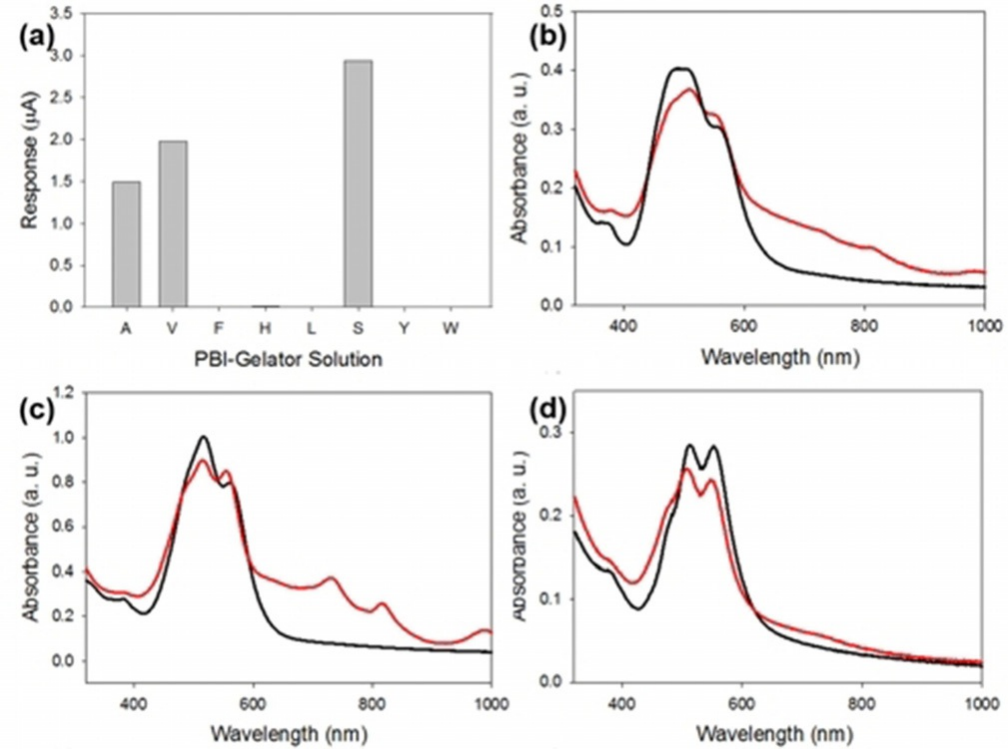
(a) Bar chart showing the photoresponse of the films at 4 V to 365 nm. The absolute values are tabulated in the Supporting Information, Table S13. (b–d) UV/Vis absorption spectra of dried solutions before irradiation (solid line) and after irradiation for 10 minutes with 365 nm LED (red line) for (b) **PBI‐A** (c) **PBI‐H** and (d) **PBI‐F**.

Differing degrees of alignment was ruled out as the reason for the varying photoresponse by aligning the structures under shear.^13^ The responses for aligned samples did not differ in magnitude from those for unaligned samples.

The UV/Vis absorption spectra of all samples showed the presence of extra peaks attributable to the radical anion after irradiation, albeit with varying intensity. **PBI‐S**, **PBI‐A**, and **PBI‐V** all showed the presence of very intense peaks due to the radical anion. These three samples also showed the presence of the dianion at 620 nm. These data could suggest that the photoresponse simply correlates with the concentration of radical anion. Alternatively, these data could suggest that the presence of dianion might be somehow connected to the higher activity of the PBI samples, although previous literature has suggested the dianion reduces the activity of PBIs.^14^

The concentration of the radical anions present in each film was measured using EPR (Figure 4 and Figure S30, Supporting Information). As for the solution data, there are significant differences in the radical anion content after irradiation, ranging from 12 % for **PBI‐A**, to 0.2 % for **PBI‐L**. The percentage of spins in both the wet and dried solution samples are generally similar, naively suggesting that the inherent propensity to generate radicals does not significantly change when going from a semi‐agglomerated solution to a dried solution, as well as showing the air‐tolerance of the radical anion. However, there are some cases where there is a significant difference. For example, there was a radical content of 6.3 % in solution of **PBI‐L**, but almost zero in the film. This implies that in some cases there are routes to quenching of the radical, presumably due to the film morphology.

**Figure 4:**
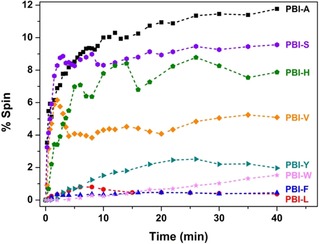
Grow in of EPR signal with time after irradiation with 365 nm light for the films of the different PBIs.

The molecular packing can be inferred to some degree from the UV/Vis spectra. From the UV/Vis data for the different PBIs in solution, **PBI‐A**, **PBI‐H**, **PBI‐V**, **PBI‐L**, and **PBI‐S** give similar data to one another (Figure S6). **PBI‐F**, **PBI‐W**, and **PBI‐Y** give data similar to each other, but differ from the previously mentioned five. In solution, there is little correlation between the UV/Vis spectra and the concentration of charges. For example, **PBI‐A** and **PBI‐Y** give similar concentrations of radical anion (9.2 % and 8.0 % respectively, see Table S12) even though they have very different initial UV/Vis spectra implying very different packing. **PBI‐F** has a very similar UV/Vis spectrum to that of **PBI‐Y**, but the concentration of radical anion is far lower (2.2 % as opposed to 8.0 %). Hence, it is not clear that there is some direct correlation between the formation and mobility of the charge and the molecular packing and it is difficult to infer from this data an optimal ideal packing on the basis of UV/Vis data.

Similarity in packing does not necessarily imply a similar self‐assembled morphology. However again there seems to be no direct correlation. For example, the SANS data for **PBI‐A** and **PBI‐L** can be fitted to a similar model (an elliptical cylinder with a radius of around 5.1 nm) but there are differences between the concentration of radical anion that can be generated (9.2 and 6.0 % respectively).

When the solutions are dried to form a film, photoconductivity requires a morphology with a continuous pathway between electrodes in addition to the generation of charge, as discussed above. These two aspects can be treated separately. From the SEM data (Figure S9), anisotropic structures are formed in the films of most of the PBIs. Only in the case of **PBI‐F** and **PBI‐W** are no such structures observed. This can therefore be used to explain the low photoconductivity for these two PBIs, compounded by the low concentration of radical anion observed on irradiation (Figure 4). For the other PBIs, it seems that the morphology would be expected to lead to continuous pathways.

In general, the concentration of the radical anion correlates with the photoconductivity, but not linearly. **PBI‐A**, **PBI‐V** and **PBI‐S** having a high concentration of spins and having the greatest photoresponse. The lowest concentration of spins was found for **PBI‐W**, **PBI‐L** and **PBI‐F**, which have the poorest photoresponse. **PBI‐H** however has a higher concentration than expected from the photoresponse measurements. Looking carefully at the data for the **PBI‐V**, there is an initial increase, followed by a decreased and then a stabilization in the concentration of the radical anion. We hypothesize that this is due to the initial formation of the radical anion, followed by formation of the dianion, which is not EPR‐active. We hypothesize therefore that, in line with the UV/Vis absorption data, the presence of the dianion is responsible for the high photoconductivity for **PBI‐A**, **PBI‐S**, and **PBI‐V** and so the high radical anion concentration for **PBI‐H** is insufficient to lead to a highly photoconductive material (the UV/Vis absorption data shown in Figure 3 c shows the absence of the dianion for **PBI‐H**). However, for the conductivity a continuous pathway is required, so morphology changes will also be playing a role. If isolated domains of the charged species are formed for example, then the increase in the radical anion observed by EPR would not correlate with increased conductivity. Hence, in an ideal system, the morphology would be optimised for both charge generation and charge transport.

Hence, for this family of PBIs, despite the predicted similarity in ionisation potential, electron affinity and ion‐pair energy for the isolated molecules, there are clear differences in the photoconductivity for the dried films. The fact that we see no correlation between this ion‐pair energy and both the radical yield and photoconductivity suggests that either some other (kinetic) factor dominates or aggregate formation results in changes of the electronic properties, similar to what we know to be true for the optical properties. If aggregate formation leads to changes in the electronic properties, it is not surprising that it does so in different ways for the different PBIs, possibly because they pack differently. The UV/Vis absorption and EPR data imply that the most photoconductive samples are those for which formation of the dianion occurs.

Overall, we have shown that the optoelectronic properties of a PBI film are not as expected from ΔDFT calculations and experimental CV measurements on the isolated molecule similar for the different PBIs but rather show a large variation. We demonstrated that this variation is likely due to the different ways in which these substituted PBIs pack together and exploited it to optimise the photoconductivity of PBI films.

Our results open the possibility for tuning the optoelectronic properties of self‐assembled PBIs by controlling the aggregate structure through erudite choice of substituent. PBIs are used in a wide range of materials, often in an aggregated, or assembled manner and we expect that our results will translate into many of these.

## Conflict of interest

The authors declare no conflict of interest.

## Supporting information

As a service to our authors and readers, this journal provides supporting information supplied by the authors. Such materials are peer reviewed and may be re‐organized for online delivery, but are not copy‐edited or typeset. Technical support issues arising from supporting information (other than missing files) should be addressed to the authors.

Supplementary
