# Supplementary material for: Controlling Photoconductivity in PBI Films by Supramolecular Assembly
Source: Chemistry. 2018 Feb 19;24(16):4006–10. doi: 10.1002/chem.201800201 (PMC5887895; doi:10.1002/chem.201800201)
Supplement: Supplementary file 1 — Supplementary [file CHEM-24-4006-s001.pdf]

CHEMISTRY
A European Journal

Supporting Information

Controlling Photoconductivity in PBI Films by Supramolecular Assembly

Emily R. Draper,*[a] Lewis J. Archibald,[a] Michael C. Nolan,[a, b] Ralf Schweins,[c]
Martijn A. Zwijnenburg,[d] Stephen Sproules,[a] and Dave J. Adams*[a]

chem_201800201_sm_miscellaneous_information.pdf

Synthesis and General Procedures

Synthesis and Characterisation

All chemicals were purchased from Sigma Aldrich or Alfa Aesar and used as received unless otherwise stated. Deionised water was used throughout.

All PBI-amino acid molecules except PBI-S were synthesised as previously reported, with the characterisation data matching that reported.[1] PBI-S had not been previously synthesised to the best of our knowledge and is reported below.

Nuclear magnetic resonance spectroscopy was carried out in deuterated dimethylsulphoxide (DMSO-d_6) for the ^{1}H NMR and in D_2O with 0.1 M LiOD for the 13C NMR due to poor solubility on the University of Glasgow Bruker Avance III HD 400 MHz spectrometer.

[N, N'-di(L-serine)-perylene-3,4:9,10-tetracarboxylic acid bisimide] (PBI-S) synthesis

In a 100 mL Schlenk flask, 1.6 g (4 mmol) of 3,4:9,10- perylenetetracarboxyldianhydride (PTCDA), 0.82 g (8 mmol) of L-serine and 2.72 g (40 mmol) of imidazole were added. These were mixed and purged with nitrogen for 10 minutes. The mixture was then heated to 120 °C under argon and left stirring for 5 hours at this temperature. The reaction was then cooled to 90 °C and 10 mL of deionised water was added. The reaction was left for an hour and then cooled to room temperature before filtering to remove unreacted PTCDA. 50 mL of 2 M HCl was then added to lower the pH to between 2 and 3, this results in a gelatinous precipitate. The precipitate was then collected by suction-filtration and washed thoroughly with acidified water. The dark red solid was dried overnight in a vacuum oven and then freeze dried to remove any remaining water. If any trace imidazole remained the PBI-S would be refluxed in acidic water for 5 hours and collected by suction filtration and again freeze-dried. All the other PBI materials were prepared in this way but have been previously reported. A typical yield obtained for all PBIs was between 80-90 %

^{1}H NMR (DMSO-d_6) 400 MHz: 12.80 (br, 2H;–OH); 8.39 (m, 4 H); 8.30 (m, 4 H); 5.72 (q, 2 H, J = 7.0 Hz); 4.15 (d, 4 H).

^{13}C NMR (0.1 M LiOD in D_2O) 100 MHz: δ (ppm) = 174.2 (COOH); 161.9 (C=O); 134.2, 133.4, 130.7, 127.8, 123.6, 121.8, 119.2 (perylene core C); 58.6 (CH); 55.7 (CH_2-OH).

Procedures

Preparation of PBI Solutions

All solutions were prepared of 5 mg/mL of gelator. For the preparation of a 2 mL solution, 10 mg of PBI was weighed into a vial. An equimolar amount of 0.1 M NaOH (aq) was added to the PBI and then was made up to 2 mL with water. The solution was then stirred using a stirrer bar overnight to ensure that all the PBI had dissolved. A large stock solution of each PBI was prepared to ensure that the solutions were the same for each experiment. All preparation of samples was done at room temperature (around 20 °C in the daytime).

Photoconductivity and Wavelength Response Measurements

All samples for photoconductivity measurements were prepared on a glass microscope slide using a 3 x 3 mm mask. This was done placing a piece of Scotch tape on the glass slide and cutting out a 3 x 3 mm square using a scalpel. The sample could then be placed in the mask and dried. Once dried the mask was removed to leave a 3 x 3 mm square of the sample. Electrodag (Agar Scientific) silver paste was then put on either side of the sample making sure that the sample was touching the sides of the film but not covering it to maintain the 3 mm gap. Copper wires were then placed into the silver paste and dried. More silver paste was then applied and again left to dry. This was done to ensure good contact with the silver. Next epoxy resin was applied over the silver paste and a little over the wire and the glass. This prevents oxidation of the silver in the air, but also makes the silver/copper wire contacts more robust and less likely to break during measurements. For the solution 10 μL of sample was placed in the mask and dried in air.

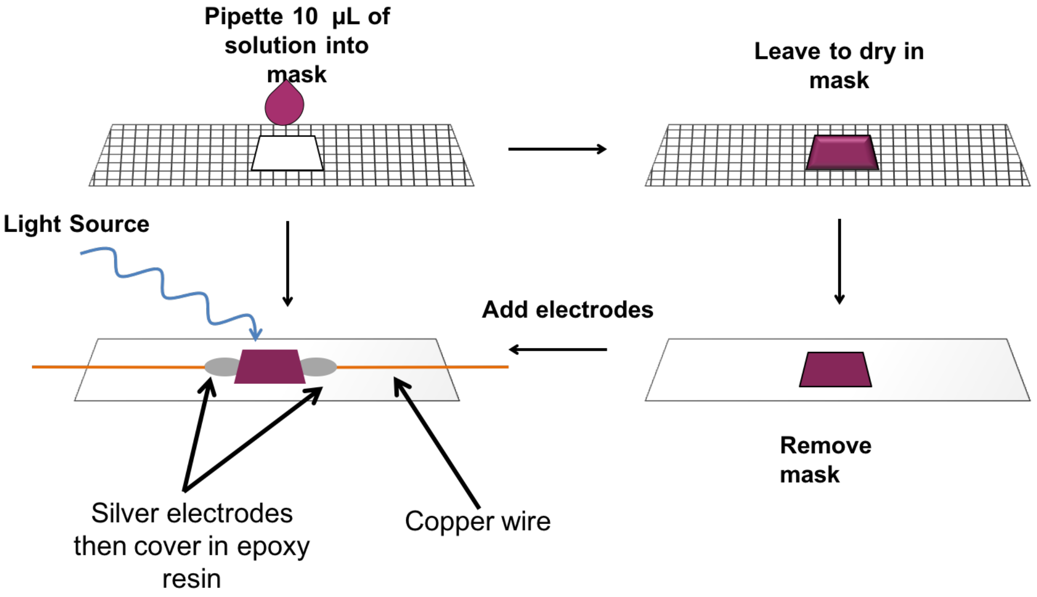

All samples for photoconductivity were prepared and measured in triplicate to ensure reproducibility and the reported values in the main text are representative of the samples. Films were imaged by cross-polarised optical microscopy to check they had not cracked or crystallised upon drying.

Conductivity and wavelength response measurements were performed as we have previously reported.[1i] Directional conductivity measurements were performed as we have previously reported.[2]

Rheological Measurements

All rheological measurements and alignment experiments were performed using an Anton Paar Physica 301 or 101 rheometer. A cone and plate geometry was used for viscosity measurements and for the shear alignment measurements. All measurements were measured in triplicate to ensure reproducibility of reported results. Measurements were carried out at 25 °C.

Viscosity measurements: Measurements were carried out using a 50 mm cone from 0.1 s^{-1} to 1000 s^{-1}. Around 1 mL of solution was placed under the cone geometry.

Alignment experiment: Alignment was carried out using a 25 mm cone geometry. A piece of glass was secured to the bottom plate and 100 µL of solution was then placed on the glass and the cone lowered on the top. A shear rate of 10 s^{-1} was then used to shear align the sample. This was left overnight for the sample to dry under shear. This method was reported in detail in our previous work.[2]

UV-Vis Absorption Spectroscopy

All UV-vis absorption spectra were collected on a Cary 60 UV-vis spectrometer from Agilent Technologies. Wet UV-vis absorption spectra were collected using a Hellma 0.1 mm demountable quartz cuvette. The gels could be formed directly in the cuvettes and then measured. Dried film samples were made by pipetting 50 µL of gelator solution onto a glass microscope slide. This was then spread over the glass to make a thinner layer of sample and allowed to dry in air. To see the formation of the radical anion, or dianion, the thin film samples were irradiated with 365 nm LED for around 10 minutes and the UV-vis absorption spectra was recorded.

Optical Microscopy and Photos

Cross-polarised light optical microscope images were taken using a Nikon LV100 Eclipse Microscope fitted with an Infinity 2 colour camera. All other photographs were taken using an iPhone 7. No post-modification or processing was made to the images after being collected.

Small Angle Neutron Scattering (SANS)

SANS measurements were performed using the D11 instrument (Institut Laue – Langevin, Grenoble, France). A neutron beam, with a fixed wavelength of 6 Å and divergence of $\Delta\lambda/\lambda$ = 9%, allowed measurements over a large range in Q [$Q = 4\pi\sin(\theta/2)/\lambda$] of 0.001 to 0.3 $Å^{-1}$, by using three sample-detector distances of 1.5 m, 8m, and 39 m. The solutions were prepared as described above, but replacing the H_2O and NaOH with D_2O and NaOD respectively. All measurements were carried out in 2 mm path length Hellma UV spectrophotometer grade, quartz cuvettes; the gels were prepared directly in these cuvettes. The cuvettes were housed in a temperature controlled sample rack during the measurements.

The data were reduced to 1D scattering curves of intensity vs. Q using the facility provided software LAMP. The electronic background was subtracted, the full detector images for all data were normalized and scattering from the empty cell was subtracted. The scattering from D_2O was also measured and subtracted from the data. The data were normalized to absolute units using a 1mm thick water sample as secondary calibration standard, with a differential scattering cross section of 0.983 1/cm for the experimental settings used. Last, data were radially averaged to produce the 1D curves for each detector position. The instrument-

independent data were then fitted to the models discussed in the text using the SasView software package version 3.1.2.[3]

Cyclic Voltammetry (CV)

CVs were collected using a three-electrode system and a Dropsens potentiostat with a glassy carbon working electrode, a Pt wire counter electrode and an Ag/AgCl reference electrode. The supporting electrolyte was 0.1 M NaCl in water and 0.1 M tetrabutylammonium hexafluoroborate (TBAHFB) in DMF. The measurements were scanned from 1.0 V to – 1.0 V, firstly 5 times at a scan rate of 0.5 V/s and a step value of 0.01 V, then 5 scans were collected at 0.05 V/s with a step of 0.005 V. The broadness and resolution of the CVs sometimes changed between scans (assumed to be due to the high viscosity of solutions) although the absolute response and potential of the peaks did not change. Therefore, the clearest scan from the 5 scans at 0.05 V/s was selected for analysis.

For calibration, the $E_{1/2}$ for the ferrocene-ferrocecium redox couple (Fc/Fc^+) in DMF was measured at 0.479 V vs. Ag/AgCl. Although not used for calibration, the $E_{1/2}$ for the ferrocene carboxylic acid redox couple ($Fc\text{-}COOH/Fc^+\text{-}COOH$) in water was measured at 0.616 V vs. Ag/AgCl.

Experimental ionisation potential (HOMO) and electron affinity (LUMO) calculations

For the determination of the electron affinity (EA)/ Lowest Unoccupied Molecular Orbital (LUMO) energy level vs. vacuum, the reduction maximum for the first reduction potential vs. Ag/AgCl was obtained from the CV data. For calculating the EA/LUMO energy in eV, potentials were then converted to vs. Fc/Fc^+ using an experimental value (+0.479 V), then to vs. SCE (+0.47 V), then to vs. SHE (-0.24 V), then to vs. vacuum (-4.28 V or -4.44 V) by using known literature conversions.[4] Therefore, the final calculation was:

$$-EA = E_{LUMO}\text{ (eV)} = (Red_{max} + 0.479\text{ V} + 0.470\text{ V} – 2.4\text{ V} – (4.28\text{ V or } 4.44\text{ V}))$$

For the aqueous solutions measured in the experiment, the solvent window for water (water oxidation) was reached at potentials > 0.9 V vs Ag/AgCl. Therefore, the oxidation of PBI expected to be around 1.5 V vs Ag/AgCl was inaccessible in aqueous solutions.

As the PBI materials in this study are used in photovoltaics, it was also deemed suitable to use the optical band gap to approximate the HOMO energy levels.

The ionisation potential (IP)/ Highest Occupied Molecular Orbital (HOMO) energy vs. vaccum was approximated by subtracting the optical band gap, E_g, from the EA/LUMO energy. E_g was calculated by using the onset of absorption from the UV-Vis spectrum (λ_{onset}) and converted to eV using the equation:

$$E_g\text{ (eV)} = 1242/\lambda$$

The approximate IP/HOMO energy was then calculated using the equation $-IP = E_{HOMO}$ (eV) $= E_{LUMO} - E_g$.

The HOMO and LUMO energies from solutions of PBI-X in both high pH water and dimethylformamide (DMF) were measured in order to compare the differences between the aggregated and dissolved material.

Theoretical calculations

The adiabatic ionisation potential (IP: PBI-XH + e^- -> PBI-XH^- or PBI-X^- + e^- -> PBI-X^{2-}) and electron affinity (EA: PBI-XH^- + e^- -> PBI-XH^{2-} or PBI-X^{2-} + e^- -> PBI-X^{3-}) of the singly- and doubly-deprotonated versions of the different PBI-X molecules (PBI-XH^-/PBI-X^{2-}), representative of the (molecular) species present at high pH, were calculated in a ΔDFT fashion using the B3LYP[5] density functional and the COSMO[6] implicit solvation model, with a dielectric permittivity value of 80. The predicted IP and EA values were subsequently converted from the vacuum scale to that of the standard hydrogen electrode (SHE) by subtracting the experimentally reported absolute value of the SHE potential, using two different values from the literature 4.44[4b] and 4.28 V[4a] respectively, and finally from the SHE scale to that of the Ag/AgCl electrode (saturated NaCl) by subtracting a further 0.197 V[7]. Ion-pair energies, the energy required to produce a pair of charged states sufficiently far separated that their electrostatic energy is negligible, also referred to as the fundamental or transport gap, are calculated from the differences of IP and EA values.

Two different basis-sets are employed; the double-ζ DZP[8] and the triple-ζ def2-TZVP[9] basis-sets, and all COSMO calculations include the outlying charge correction, which is found to change potentials by typically less than 0.1 V. All potential and ion-pair energy calculations, finally, are performed using Turbomole[10] 7.01.

SEM Imaging

High resolution imaging of the PBI hydrogel morphology was obtained using a Hitachi S-4800 FE SEM. PBI hydrogels were deposited onto conductive glass slides and attached to 15 mm Hitachi M4 aluminium stubs using an adhesive high-purity carbon tab before and air dried. The PBI samples were gold coated with gold for 1 minute at 25 mA a Emitech K550X automated sputter coater. Imaging was carried out at a working distance of 9 mm with an applied working voltage of 3 kV using a mix of upper and lower secondary electron detectors. The FE-SEM measurement scale bar was calibrated using certified SIRA calibration standards.

EPR Spectroscopy

All EPR data were recorded at X-band frequency (9.67 GHz) on a Bruker ELEXSYS E500 spectrometer equipped with an ER 4102ST-O optical transmission resonator. All measurements were collected on 0.5 mg/mL aqueous solutions in 1 mm o.d. quartz tubes. Film samples were deposited on 15 × 8 × 0.5 mm quartz plates and mounted in the middle of the resonator using a homemade sample holder. The holder was rotated 90° between irradiation and EPR data collection at various time intervals over 40 min. Field-swept spectra represent 5 scan averages collected over a 5 mT sweep width centered at 344.8 mT, with

modulation frequency = 100 kHz, modulation amplitude = 0.05 mT, receiver gain = 60 dB, time constant = 40.96 s, sampling time = 10.24 s, and microwave power = 0.63 mW. Timescan measurements were performed at a fixed field position corresponding to the peak maximum in the first derivative spectrum under continuous irradiation with a 365 nm LED. Spin counts of solution samples were quantified by double integration of the first derivative spectrum and calibrated to a 0.5 mg mL^{-1} aqueous solution of TEMPO recorded under identical conditions. For solid state spectra (film) were curve fitted, and the fit double integrated to get the signal intensity for each time interval. This was converted to a spin count by comparison to the weak pitch standard measured under identical conditions.

Solution samples for EPR were described as previously mentioned and transferred into soda glass capillary tubes with a diameter of 2 mm. Tubes were sealed at one end and samples filled 2 cm of the capillary tube.

Samples for solid EPR were prepared on pieces of 1 mm thick glass which was 8 mm by 20 mm. This could then be mounted inside the EPR spectrometer. The samples were then prepared the same as for photoconductivity measurements using a 3 x 3 mm mask. 10 μL of sample was pipetted into the mask and left to dry before the mask was removed.

Both solution and dried samples were irradiated with a 365 nm LED from LedEngin with an IsoTech CD laboratory power supply at 0.7 A and 3.7 V. Samples were irradiated until the signal intensity became stable.

Supplementary Figures

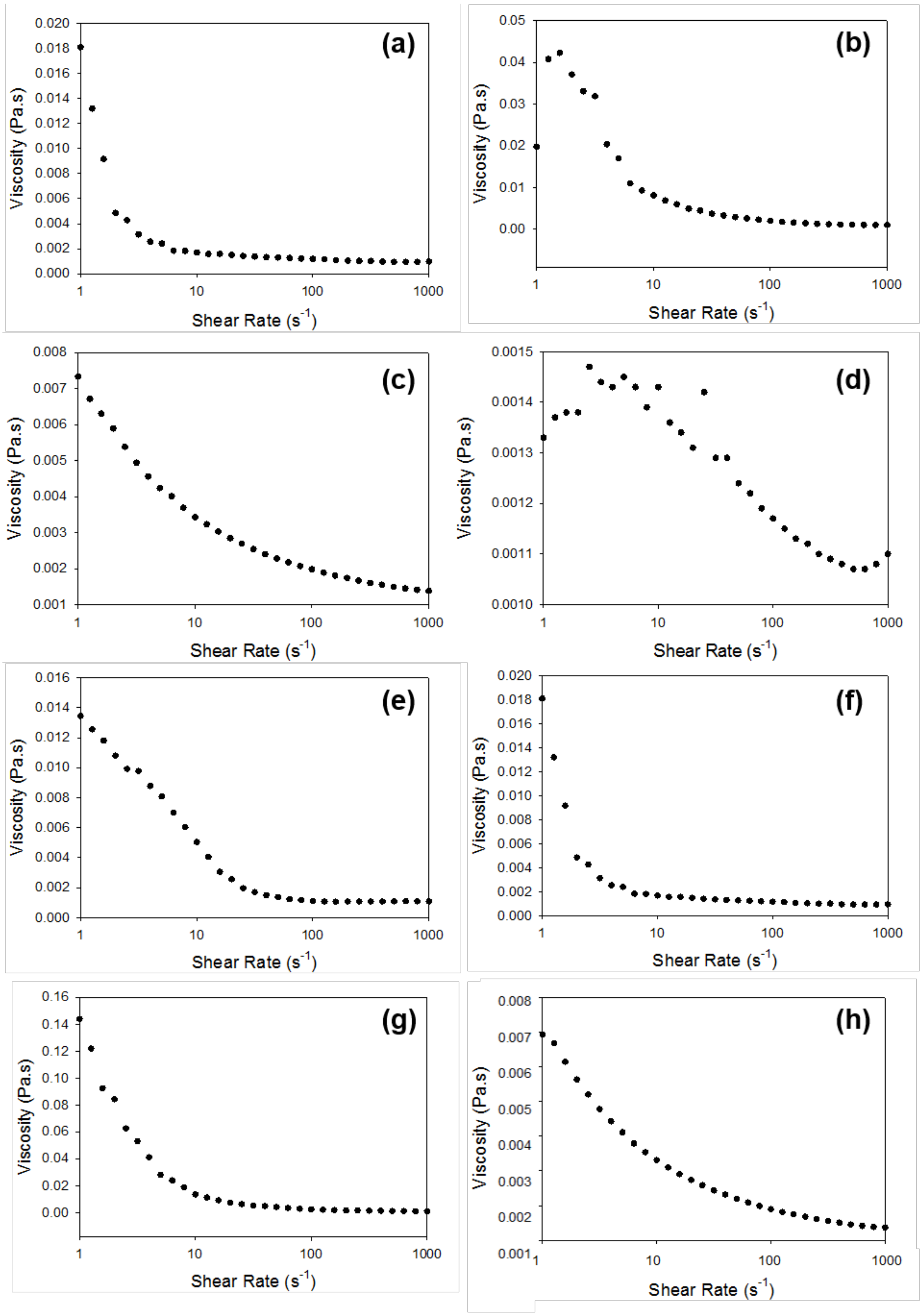

Figure S1. Dynamic viscosity measurements of (a) **PBI-A**, (b) **PBI-F**, (c) **PBI-H**, (d) **PBI-L**, (e) **PBI-S**, (f) **PBI-V**, (g) **PBI-W** and (h) **PBI-Y** at pH 8 and a concentration of 5 mg/mL.

Table S1. Summary of fits to the SANS data for the PBI solutions at high pH that could be fit to a power law model (available in SASView) alone.

	**PBI-V**	**PBI-W**	**PBI-Y**
**Power Law**	2.20 ± 0.04	3.46 ± 0.07	3.09 ± 0.01
χ^2	3.47	6.34	7.51

Table S2. Summary of fits to the SANS data for the PBI solutions at high pH that could be fit to a power law in combination with a Kratky-Porod flexible cylinder model (available in SASView).

	**PBI-F**	**PBI-H**
**Power Law**	3.42 ± 0.02	2.31 ± 0.01
**Kuhn Length / Å**	36.4 ± 2.72	75.4 ± 10.4
**Length / Å**	> 1000	670.4 ± 340.4
**Radius / Å**	10.9 ± 0.8	71.5 ± 2.1
χ^2	1.6776	2.84

Table S3. Summary of fits to the SANS data for the PBI solutions at high pH that could be fit to a power law in combination with a flexible elliptical cylinder model (available in SASView).

	**PBI-A**	**PBI-L**	**PBI-S**
**Power Law**	2.32 ± 0.02	2.30 ± 0.05	2.47 ± 0.07
**Axis Ratio**	1.96 ± 0.01	2.95 ± 0.03	1.90 ± 0.09
**Kuhn Length / Å**	280.2 ± 35.1	215.6 ± 8.8	261.5 ± 81.1
**Length / Å**	> 1000	> 1000	> 1000
**Radius / Å**	50.9 ± 0.2	51.3 ± 0.4	96.1 ± 1.8
χ^2	2.2984	2.689	2.2916

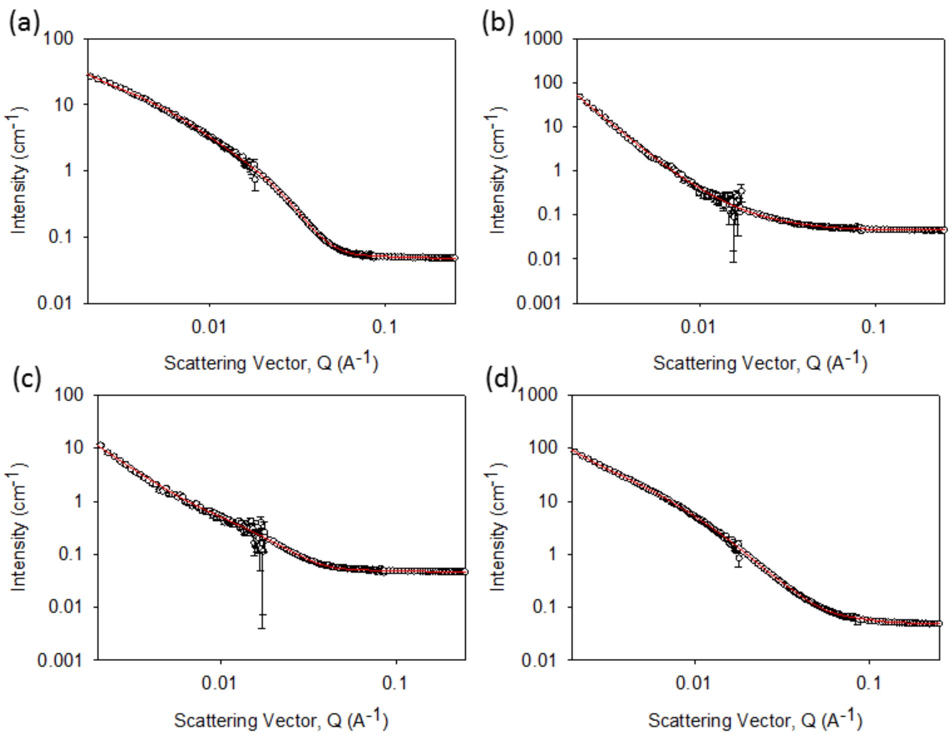

Figure S2. (a) SANS data (open circles) and fit to data (red line) for solutions of (a) **PBI-A**; (b) SANS data (open circles) and fit to data (red line) for **PBI-F**; (c) SANS data (open circles) and fit to data (red line) for **PBI-H**; (d) SANS data (open circles) and fit to data (red line) for **PBI-L**.

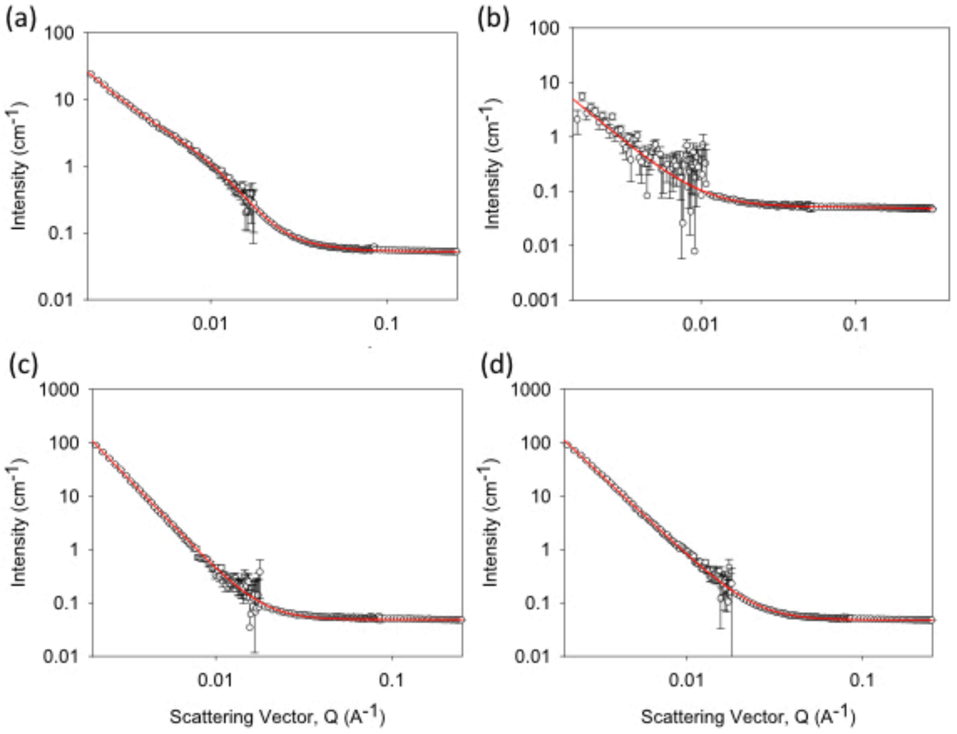

Figure S3. (a) SANS data (open circles) and fit to data (red line) for solutions of (a) **PBI-S**; (b) SANS data (open circles) and fit to data (red line) for **PBI-V**; (c) SANS data (open circles) and fit to data (red line) for **PBI-W**; (d) SANS data (open circles) and fit to data (red line) for **PBI-Y**.

Table S4. Calculation of the optical band gap using the UV-vis absorption spectra at high pH.

Gelator	λ_{max} S_0-S_1 0-0 (nm)	λ_{onset} (nm)	Optical Band Gap, E_g (eV)
**PBI-A**	542	690	1.80
**PBI-F**	548	697	1.78
**PBI-H**	552	698	1.78
**PBI-L**	548	702	1.77
**PBI-S**	545	678	1.83
**PBI-V**	543	696	1.78
**PBI-W**	550	703	1.77
**PBI-Y**	549	692	1.80

Table S5. Ionization potential and electron affinity values measured using CV for the solutions at high pH.

Gelator	$Reduction_{max}$ (V vs. Ag/AgCl)	$Oxidation_{max}$ (V vs. Ag/AgCl)	$E_{1/2}$ (V vs. Ag/AgCl)
**PBI-A**	-0.62	-0.50	-0.56
**PBI-F**	-0.72	-0.50	-0.61
**PBI-H**	-0.62	-0.39	-0.50
**PBI-L**	-0.53	-0.41	-0.47
**PBI-S**	-0.59	-0.48	-0.53
**PBI-V**	-0.64	-0.49	-0.57
**PBI-W**	-0.63	-0.49	-0.56
**PBI-Y**	-0.57	-0.43	-0.50

Table S6. Ionization potential (IP) and electron affinity (EA)values calculated for the solutions at high pH.

Gelator	-EA (eV vs. vacuum)		-IP(eV vs. vacuum)	
	High pH[a]	High pH[b]	High pH[a]	High pH[b]
**PBI-A**	-3.91	-4.07	-5.72	-5.88
**PBI-F**	-3.96	-4.12	-5.74	-5.90
**PBI-H**	-3.86	-4.02	-5.64	-5.80
**PBI-L**	-3.82	-3.98	-5.59	-5.75
**PBI-S**	-3.89	-4.05	-5.72	-5.88
**PBI-V**	-3.92	-4.08	-5.71	-5.87
**PBI-W**	-3.91	-4.07	-5.68	-5.84
**PBI-Y**	-3.85	-4.01	-5.65	-5.81

[a] using the value SHE vs. vacuum = 4.28 eV[4a]

[b] using the value SHE vs. vacuum = 4.44 eV[4b]

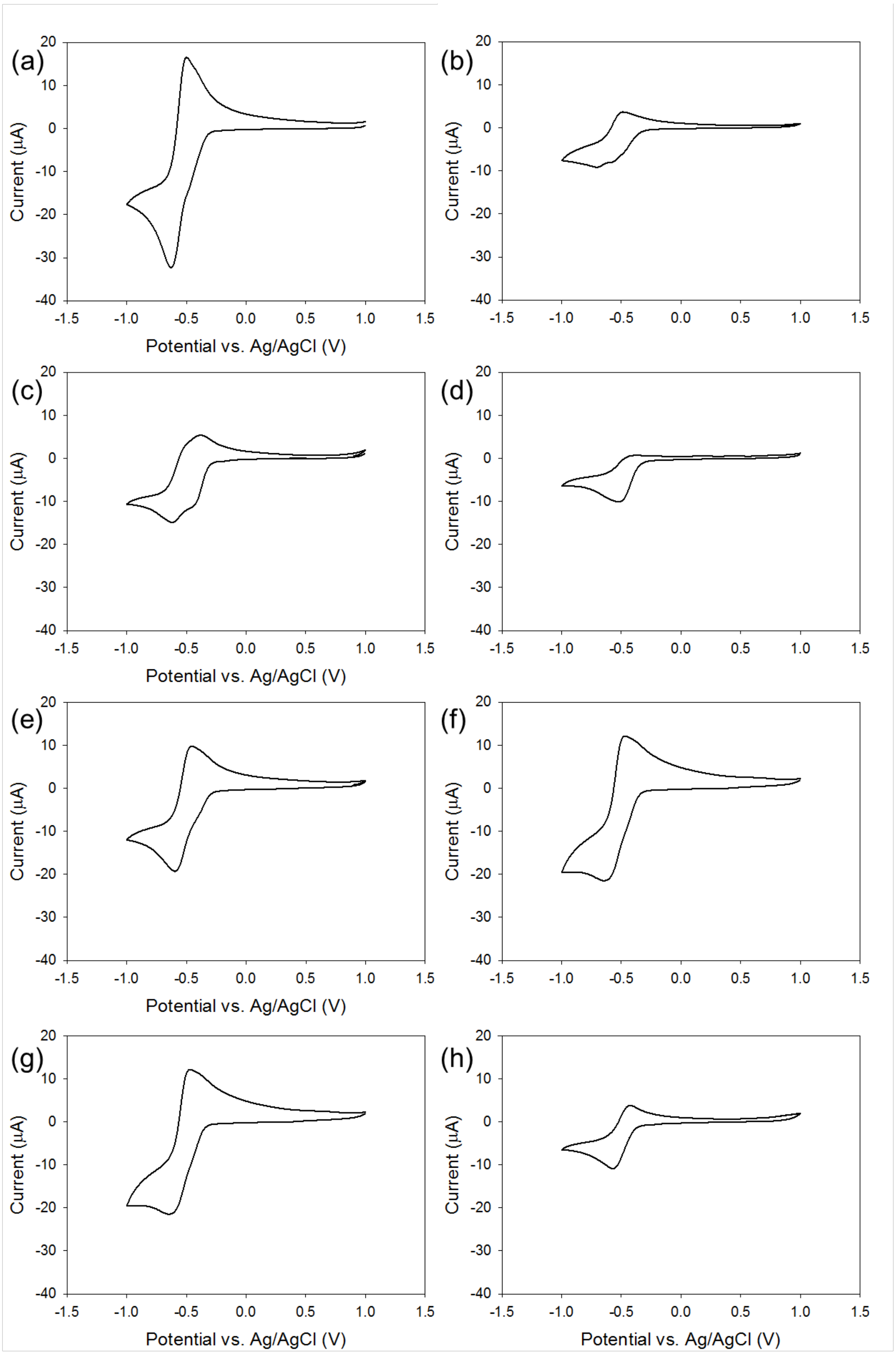

Figure S4. Cyclic voltammagram of high pH solutions of (a) **PBI-A**, (b) **PBI-F**, (c) **PBI-H**, (d) **PBI-L**, (e) **PBI-S**, (f) **PBI-V**, (g) **PBI-W** and (h) **PBI-Y**. Graphs have been scaled to the same values to compare all the data.

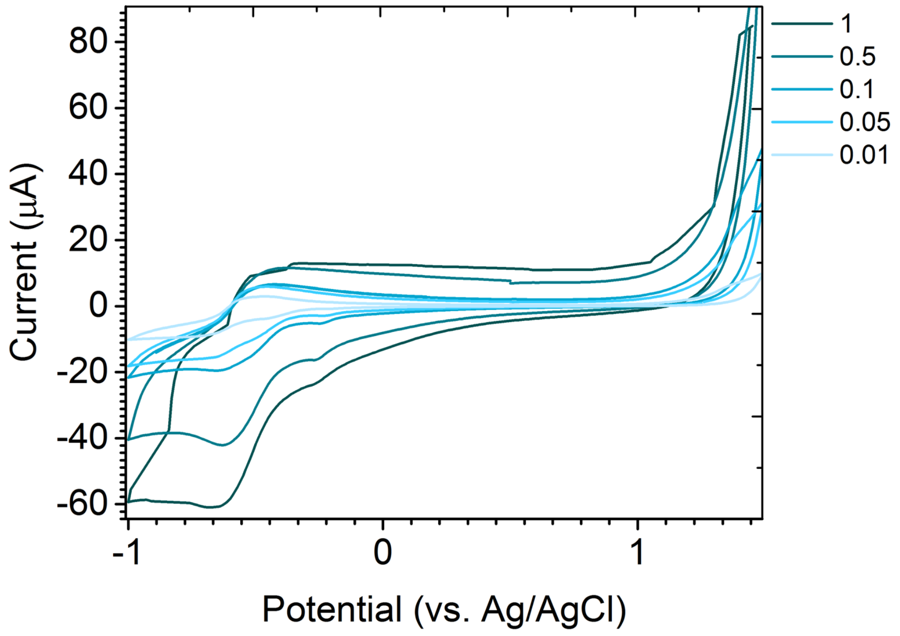

Figure S5. Cyclic voltammatry of high pH solution of **PBI-A** at different scan rates.

Table S7. B3LYP/DZP and B3LYP/def2-TZVP predicted redox potentials for the different doubly deprotonated molecules in water on the vacuum scale and ion-pair energies ($E_{ion\ pair}$), the energy required to make a pair of charge-separated states. All values in (electron)Volt.

	-IP (cation)	-EA(anion)	$E_{ion\ pair}$	-IP(cation)	-EA(anion)	$E_{ion\ pair}$
	DZP			def2-TZVP		
**PBI-A**	-5.43	-3.68	1.75	-6.02	-3.86	2.16
**PBI-F**	-5.51	-3.68	1.83	-6.06	-3.87	2.20
**PBI-H**	-5.46	-3.66	1.80	-6.03	-3.85	2.18
**PBI-L**	-5.45	-3.69	1.76			
**PBI-S**	-5.47	-3.69	1.78			
**PBI-V**	-5.41	-3.67	1.74			
**PBI-W**	-5.09	-3.67	1.42			
**PBI-Y**	-5.49	-3.67	1.82			

Table S8. B3LYP/DZP and B3LYP/def2-TZVP predicted redox potentials for the different doubly deprotonated molecules in water versus the Ag/AgCl electrode (saturated NaCl). Values before and after the slash differ in the experimental value of the absolute value of the standard hydrogen electrode potential used, 4.44 and 4.28 V respectively, where the values in the table are calculated as $E_{Ag/AgCl} = E_{vacuum} - 4.44/4.28 - 0.197$. All values in (electron)Volt.

	IP (cation)	EA(anion)	IP(cation)	EA(anion)
	DZP		def2-TZVP	
**PBI-A**	0.79/0.95	-0.96/-0.80	1.38/1.54	-0.78/-0.62
**PBI-F**	0.87/1.03	-0.96/-0.80	1.43/1.59	-0.77/-0.61
**PBI-H**	0.83/0.99	-0.97/-0.81	1.40/1.56	-0.78/-0.62
**PBI-L**	0.81/0.97	-0.95/-0.79		
**PBI-S**	0.84/1.00	-0.94/-0.78		
**PBI-V**	0.77/0.93	-0.96/-0.80		
**PBI-W**	0.45/0.61	-0.97/-0.81		
**PBI-Y**	0.86/1.02	-0.96/-0.80		

Table S9. B3LYP/DZP and B3LYP/def2-TZVP predicted redox potentials for the different singly-deprotonated molecules in water on the vacuum scale and ion-pair energies ($E_{ion\ pair}$), the energy required to make a pair of charge-separated states. All values in (electron)Volt.

	-IP (cation)	-EA(anion)	$E_{ion\ pair}$	-IP(cation)	-EA(anion)	$E_{ion\ pair}$
	DZP			def2-TZVP		
**PBI-A**	-5.40	-3.55	1.85	-5.99	-3.75	2.23
**PBI-F**	-5.48	-3.55	1.94	-6.05	-3.76	2.29
**PBI-H**	-5.43	-3.54	1.89	-6.01	-3.75	2.26
**PBI-L**	-5.42	-3.55	1.86			
**PBI-S**	-5.44	-3.56	1.89			
**PBI-V**	-5.39	-3.54	1.84			
**PBI-W**	-5.05	-3.54	1.51			
**PBI-Y**	-5.43	-3.55	1.91			

Table S10. B3LYP/DZP and B3LYP/def2-TZVP predicted redox potentials for the different singly- deprotonated molecules in water versus the Ag/AgCl electrode (saturated NaCl). Values before and after the slash differ in the experimental value of the absolute value of the standard hydrogen electrode potential used, 4.44 and 4.28 V respectively, where the values in the table are calculated as $E_{Ag/AgCl} = E_{vacuum}$– 4.44/4.28 – 0.197. All values in (electron)Volt.

	IP (cation)	EA(anion)	IP(cation)	EA(anion)
	DZP		def2-TZVP	
**PBI-A**	0.77/0.93	-1.08/ -0.93	1.35/ 1.51	-0.88/ -0.72
**PBI-F**	0.85/ 1.01	-1.09/ -0.93	1.41/ 1.57	-0.88/ -0.72
**PBI-H**	0.80/ 0.96	-1.10/ -0.94	1.38/ 1.54	-0.88/ -0.72
**PBI-L**	0.78/ 0.94	-1.08/ -0.92		
**PBI-S**	0.81/ 0.97	-1.08/-0.92		
**PBI-V**	0.75/ 0.91	-1.10/ -0.94		
**PBI-W**	0.41/ 0.57	-1.10/ -0.94		
**PBI-Y**	0.82/ 0.98	-1.09/ -0.93		

Tables S7-S10 gives the IP, EA and ion-pair energies predicted for the different singly and doubly deprotonated PBIs as calculated with B3LYP/DZP and B3LYP/def2-TZVP. Calculations with both basis-sets give similar results and display a similar trend, more about which below, but use of the larger def2-TZVP basis-set, which for reasons of computational tractability has only been applied to a subset of molecules, results in the prediction of deeper IP values, slightly more shallow EA values and slightly larger ion-pair energies. Focussing on the def2-TZVP results, there is also a decent fit to the experimentally measured EA values under high pH conditions in table SX, as well as the experimental estimates of the IP values obtained by subtracting from the measured EA the adiabatic S_1-S_0 0-0 excitation energy, extracted from the UV-Vis absorption spectra.

The calculations predict that the IP, EA and ion-pair energy values are very similar for the different singly- and doubly-deprotonated PBI-X molecules. A similar lack of change when varying the amino acid substituents is predicted for neutral PBI-X molecules in water (see Table S11). The differences in radical yield observed by EPR and UV-Vis between the different PBIs in solution is thus unlikely to be the result of differences in the fundamental electronic properties of the molecules.

Table S11. *B3LYP/DZP and B3LYP/def2-TZVP predicted redox potentials for the different neutral molecules in water on the vacuum scale and ion-pair energies ($E_{ion\ pair}$), the energy required to make a pair of charge-separated states. All values in (electron)Volt.*

	-IP (cation)	-EA(anion)	$E_{ion\ pair}$
	DZP		
**PBI-A**	-5.79	-3.80	1.99
**PBI-F**	-5.82	-3.81	2.01
**PBI-H**	-5.80	-3.79	2.01
**PBI-L**	-5.82	-3.82	2.00
**PBI-S**	-5.81	-3.82	1.99
**PBI-V**	-5.79	-3.80	1.99
**PBI-W**	-5.81	-3.80	2.01
**PBI-Y**	-5.39	-3.79	1.60

The main difference between the neutral and the singly-/doubly-deprotonated cases is the localization of the hole. In line with what we observed in previous work for PBI-A[11], the hole localizes, like an additional electron, on the PBI core in the neutral case and on the carboxylic acid group of the amino acid group after (partial) deprotonation. The only exception is PBI-Y, where both in the neutral and doubly deprotonated case, the hole localizes on the indole side-chain of the tryptophan amino acid substituent.

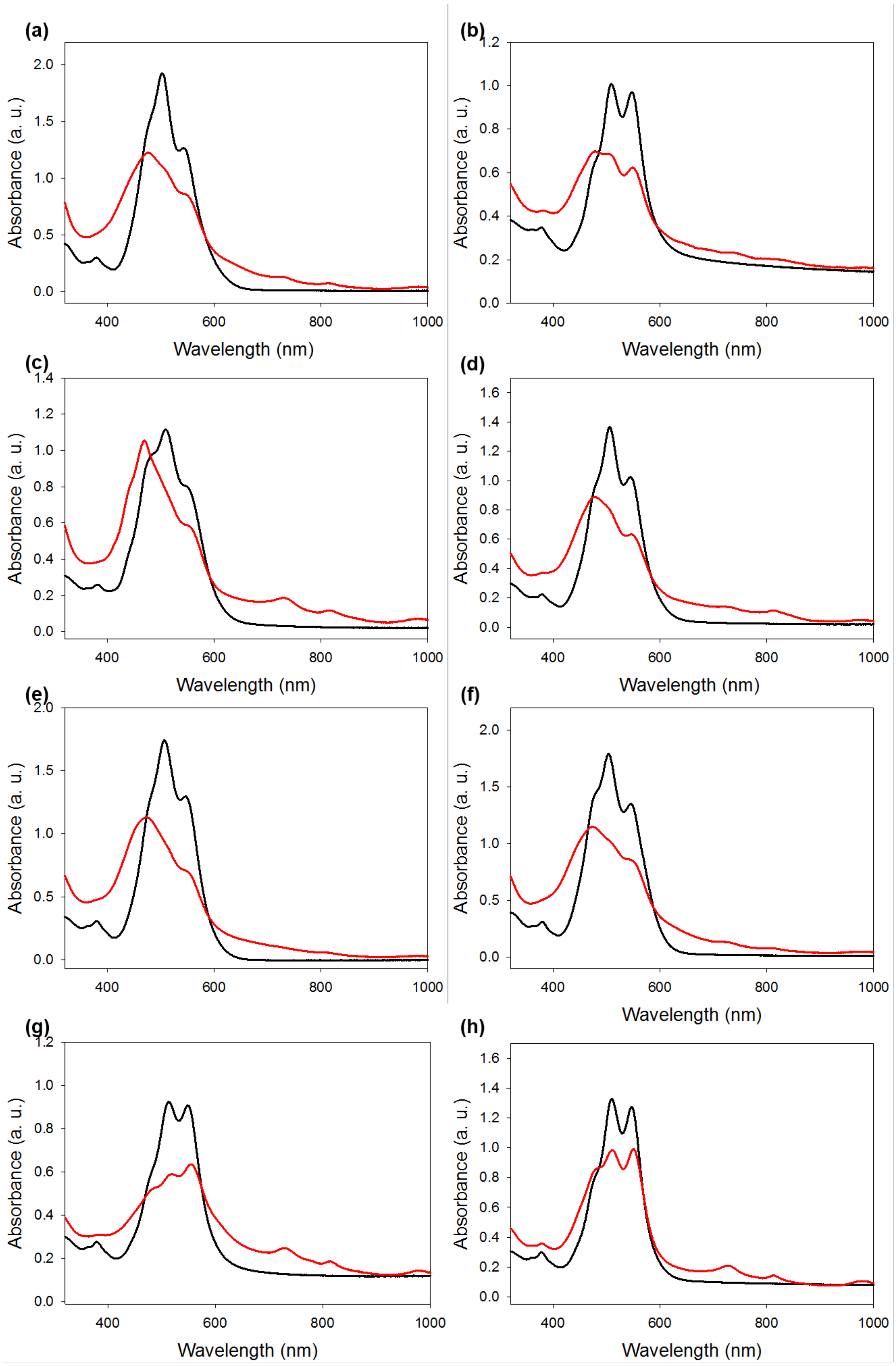

Figure S6. UV-Vis absorption spectrum in high pH solution at 5 mg/mL in a 0.1 mm quartz cuvette for (a) **PBI-A**, (b) **PBI-F**, (c) **PBI-H**, (d) **PBI-L**, (e) **PBI-S**, (f) **PBI-V**, (g) **PBI-W** and (h) **PBI-Y**. The black data is before irradiation and the red date is after irradiation with a 365 nm LED.

Table S12. Percent of radical present in the PBI solution determined by EPR after irradiation with 365 nm.

	**PBI-A**	**PBI-F**	**PBI-H**	**PBI-L**	**PBI-S**	**PBI-V**	**PBI-W**	**PBI-Y**
**% of radical present**	9.2	2.2	9.3	6.3	9.0	12.5	3.2	8.0
***g*-value**	2.0034	2.0032	2.0032	2.0033	2.0033	2.0034	2.0033	2.033

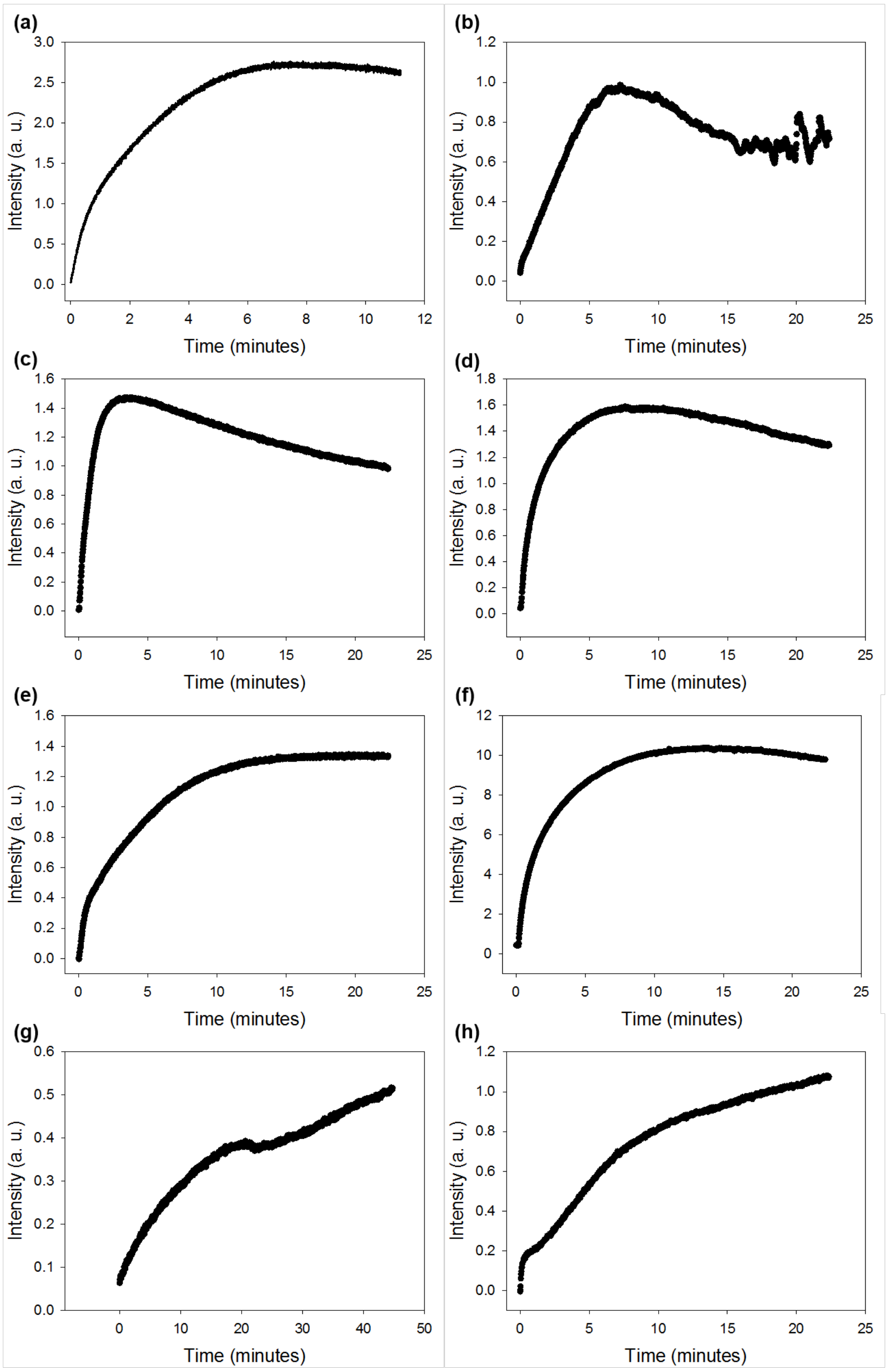

Figure S7. Grow in peak for the radical anion in the EPR spectrum after turn on in light high pH solution at 5 mg/mL in soda glass capillary tube for (a) **PBI-A**, (b) **PBI-F**, (c) **PBI-H**, (d) **PBI-L**, (e) **PBI-S**, (f) **PBI-V**, (g) **PBI-W** and (h) **PBI-Y**.

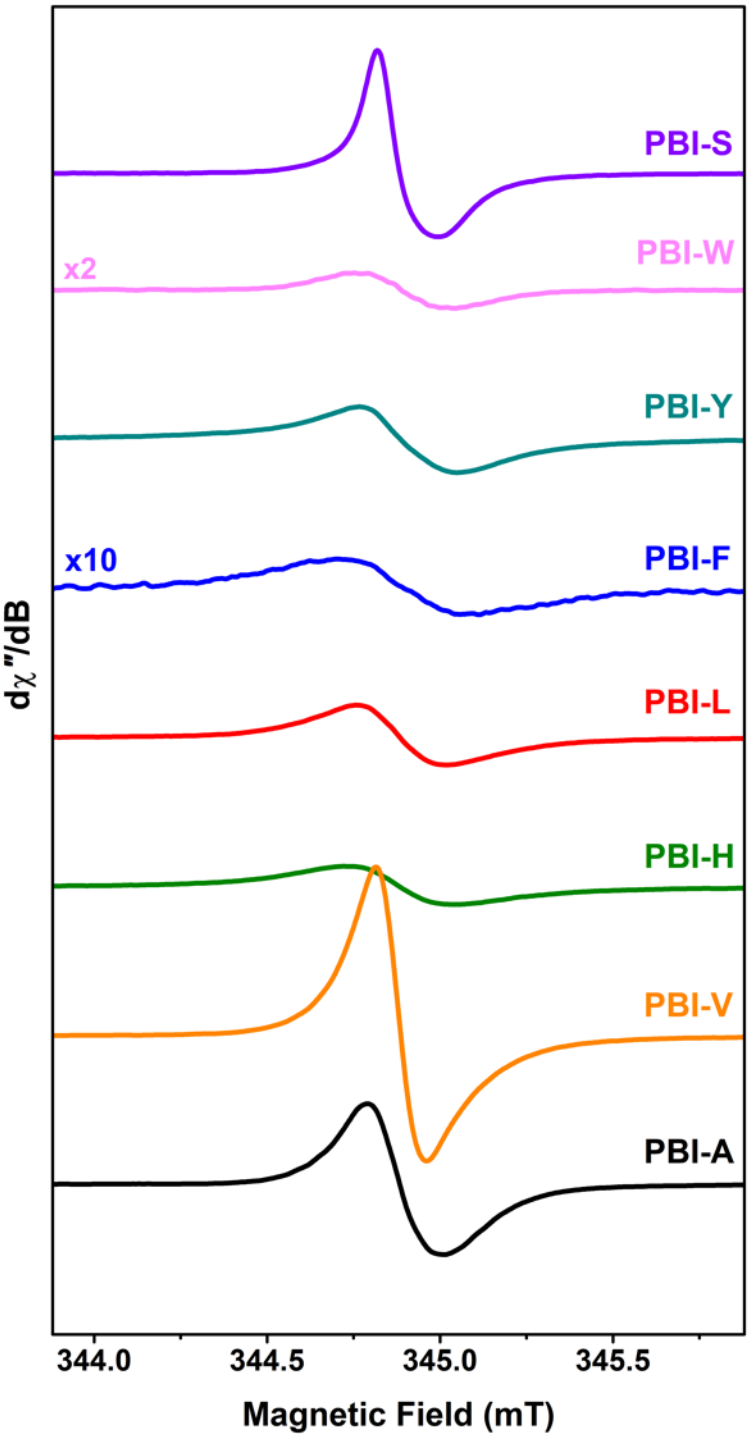

Figure S8. Comparison of the normalized X-band EPR spectra of the PBIs in aqueous solution at ambient temperature after achieving maximum signal intensity post irradiation with 365 nm (experimental conditions: frequency, 9.67 GHz; power, 0.63 mW; modulation, 0.05 mT).

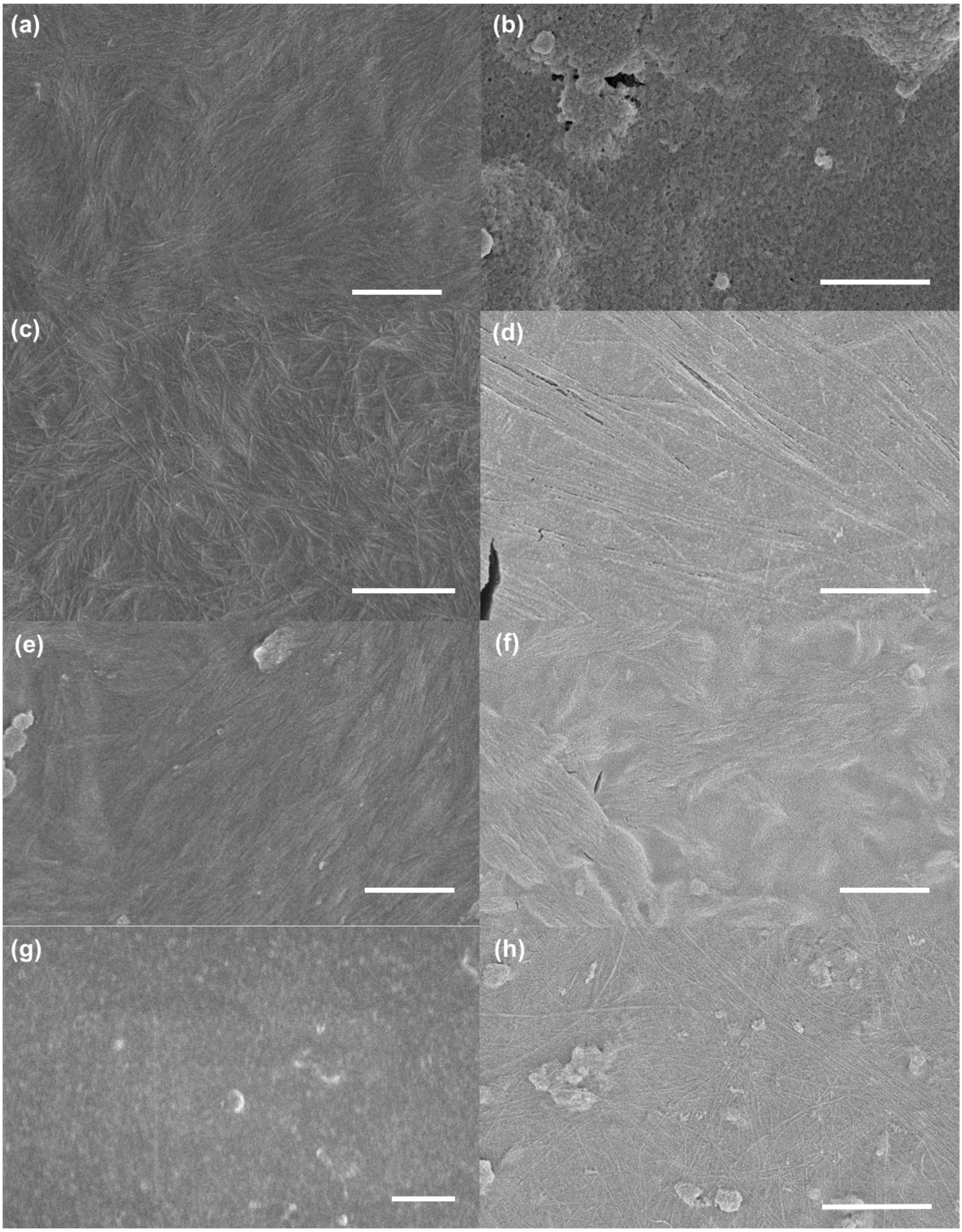

Figure S9. SEM of the dried samples of (a) **PBI-A**, (b) **PBI-F**, (c) **PBI-H**, (d) **PBI-L**, (e) **PBI-S**, (f) **PBI-V**, (g) **PBI-W** and (h) **PBI-Y**. Scale bar represens 100 nm on all images.

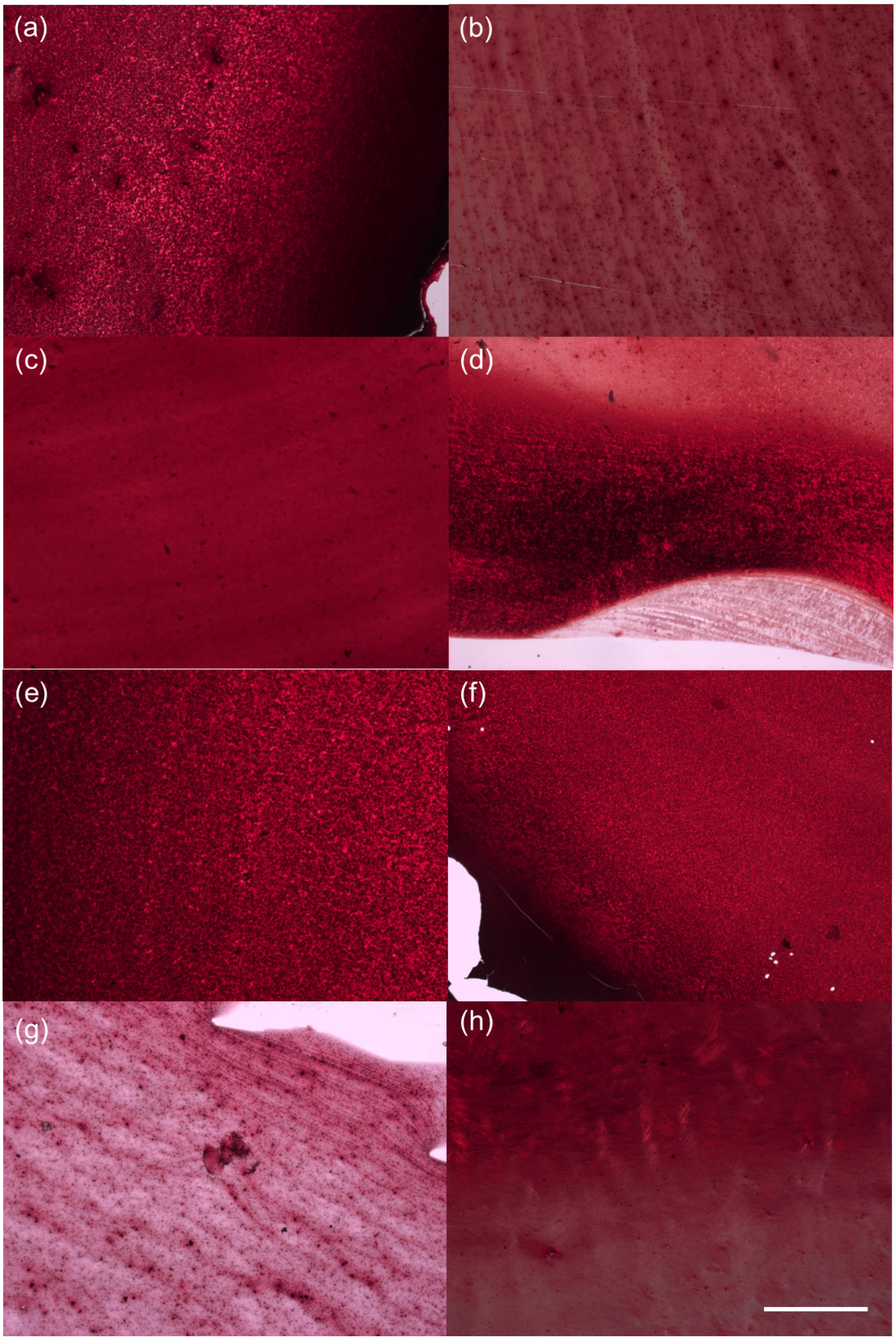

Figure S10. Cross-polarised light microscope images of dried films of (a) **PBI-A**, (b) **PBI-F**, (c) **PBI-H**, (d) **PBI-L**, (e) **PBI-S**, (f) **PBI-V**, (g) **PBI-W** and (h) **PBI-Y**. Scale bar respresents 50 μm.

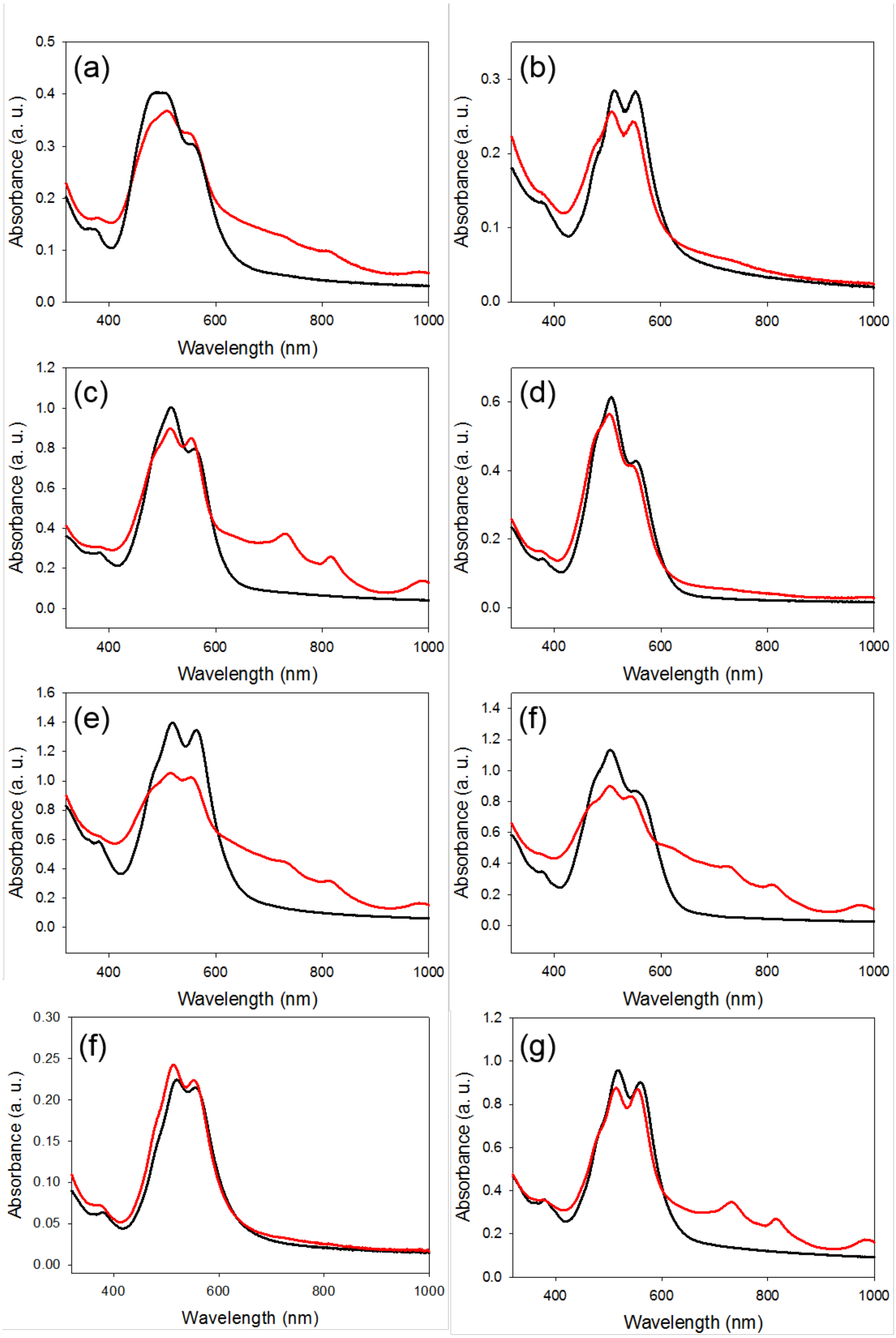

Figure S11. UV-Vis absorption spectrum in high pH dried solution at 5 mg/mL on glass for (a) **PBI-A**, (b) **PBI-F**, (c) **PBI-H**, (d) **PBI-L**, (e) **PBI-S**, (f) **PBI-V**, (g) **PBI-W** and (h) **PBI-Y**. The black data is before irradiation and the red data is after irradition for 10 minutes with 365 nm LED.

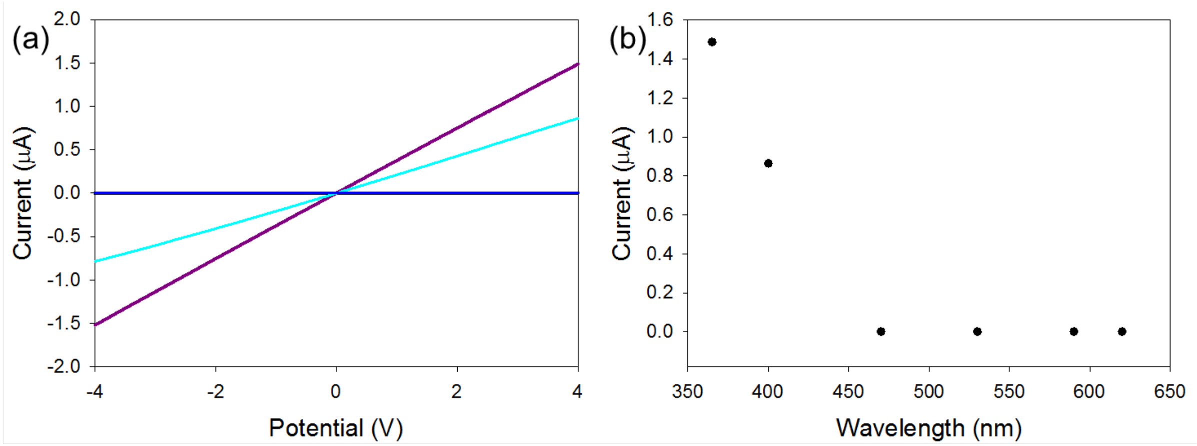

Figure S12. Photoresponse using different wavelengths of light for dried solution of **PBI-A**. (a) I-V curves in the dark (black line), after 365 nm (purple line), 400 nm (light blue line), 450 nm (blue line), 528 nm (green line), 590 nm (yellow line) and 620 nm (red line). (b) Scatter graph showing the wavelength preference of the dried solution of **PBI-A**.

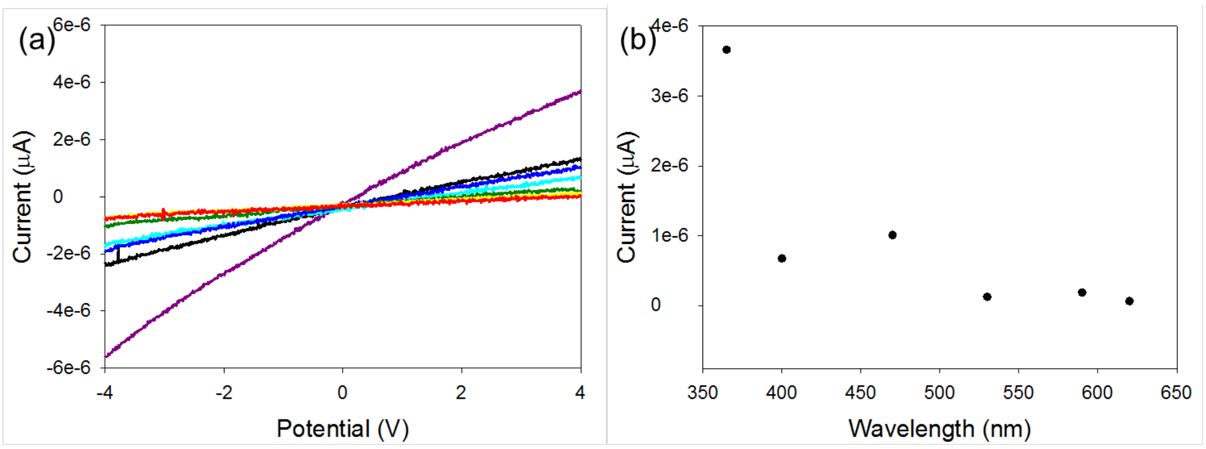

Figure S14. Photoresponse using different wavelengths of light for dried solution of **PBI-F**. (a) I-V curves in the dark (black line), after 365 nm (purple line), 400 nm (light blue line), 450 nm (blue line), 528 nm (green line), 590 nm (yellow line) and 620 nm (red line). (b) Scatter graph showing the wavelength preference of the dried solution of **PBI-F**.

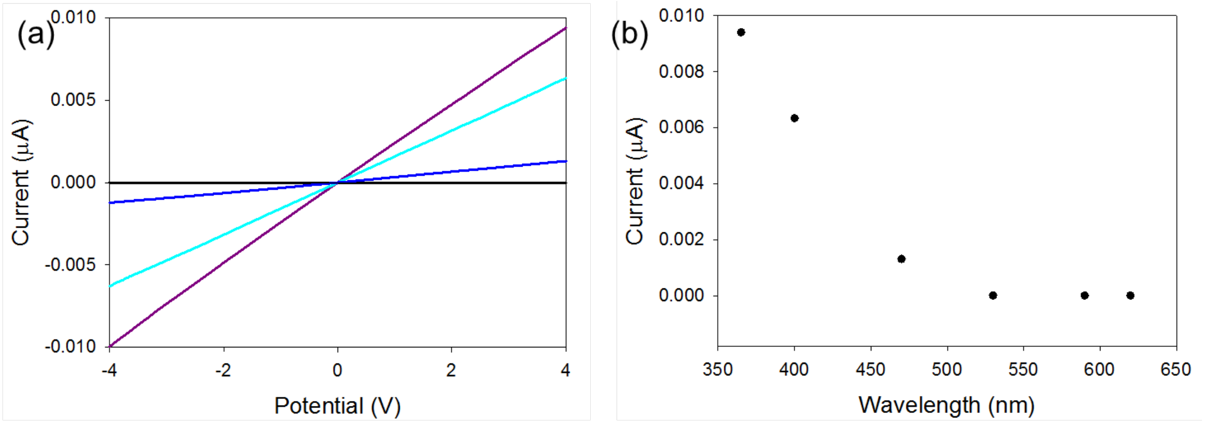

Figure S15. Photoresponse using different wavelengths of light for dried solution of **PBI-H**. (a) I-V curves in the dark (black line), after 365 nm (purple line), 400 nm (light blue line), 450 nm (blue line), 528 nm (green line), 590 nm (yellow line) and 620 nm (red line). (b) Scatter graph showing the wavelength preference of the dried solution of **PBI-H**.

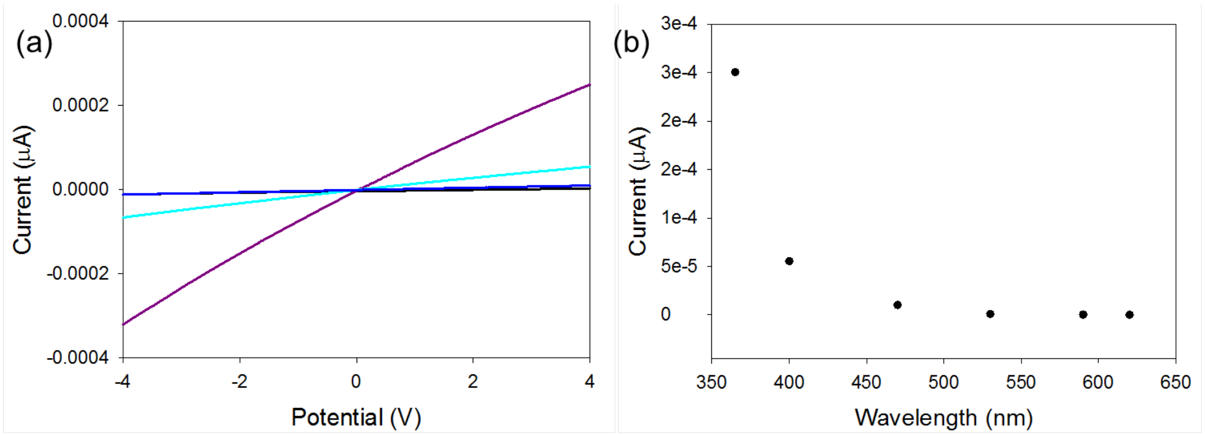

Figure S16. Photoresponse using different wavelengths of light for dried solution of **PBI-L**. (a) I-V curves in the dark (black line), after 365 nm (purple line), 400 nm (light blue line), 450 nm (blue line), 528 nm (green line), 590 nm (yellow line) and 620 nm (red line). (b) Scatter graph showing the wavelength preference of the dried solution of **PBI-L**.

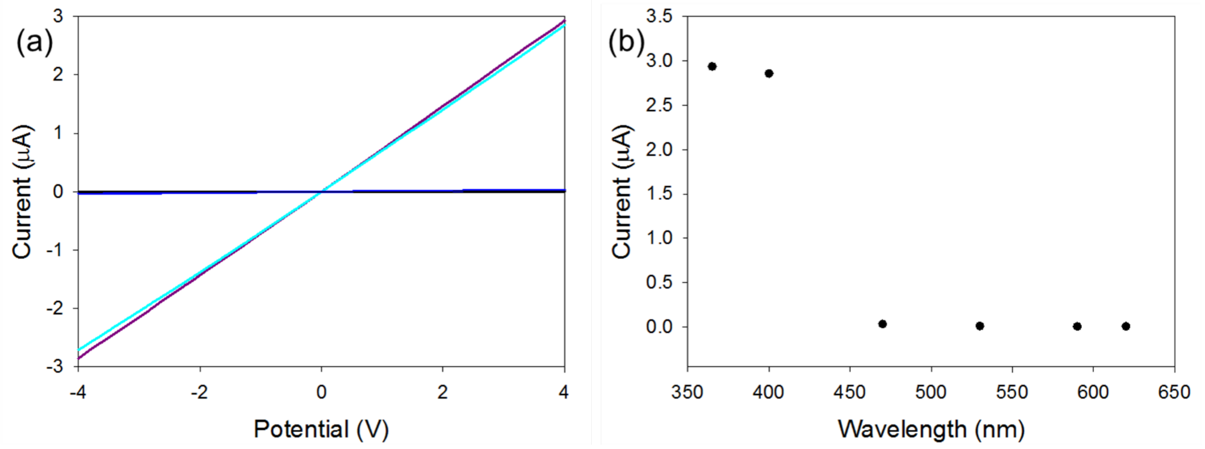

Figure S17. Photoresponse using different wavelengths of light for dried solution of **PBI-S**. (a) I-V curves in the dark (black line), after 365 nm (purple line), 400 nm (light blue line), 450 nm (blue line), 528 nm (green line), 590 nm (yellow line) and 620 nm (red line). (b) Scatter graph showing the wavelength preference of the dried solution of **PBI-S**.

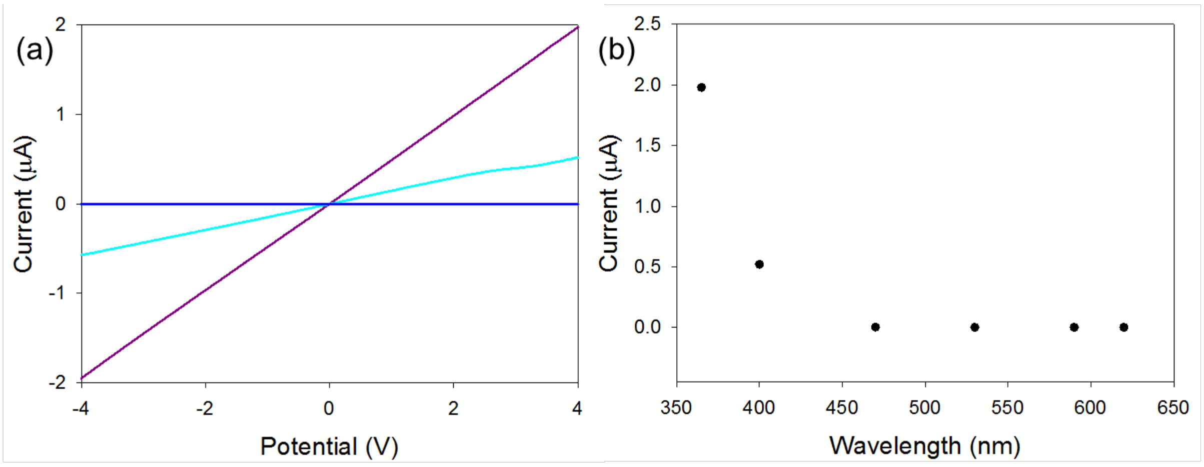

Figure S18. Photoresponse using different wavelengths of light for dried solution of **PBI-V**. (a) I-V curves in the dark (black line), after 365 nm (purple line), 400 nm (light blue line), 450 nm (blue line), 528 nm (green line), 590 nm (yellow line) and 620 nm (red line). (b) Scatter graph showing the wavelength preference of the dried solution of **PBI-V**.

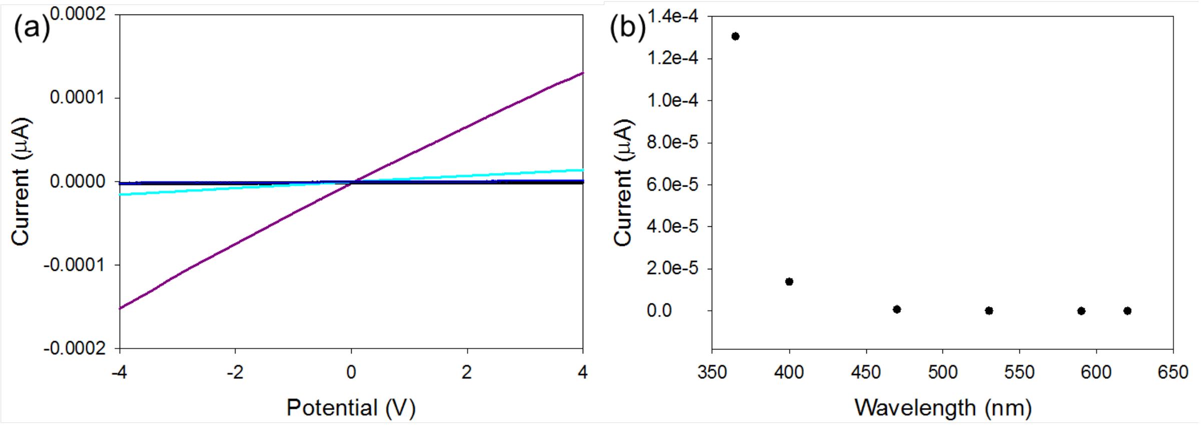

Figure S19. Photoresponse using different wavelengths of light for dried solution of **PBI-Y**. (a) I-V curves in the dark (black line), after 365 nm (purple line), 400 nm (light blue line), 450 nm (blue line), 528 nm (green line), 590 nm (yellow line) and 620 nm (red line). (b) Scatter graph showing the wavelength preference of the dried solution of **PBI-Y**.

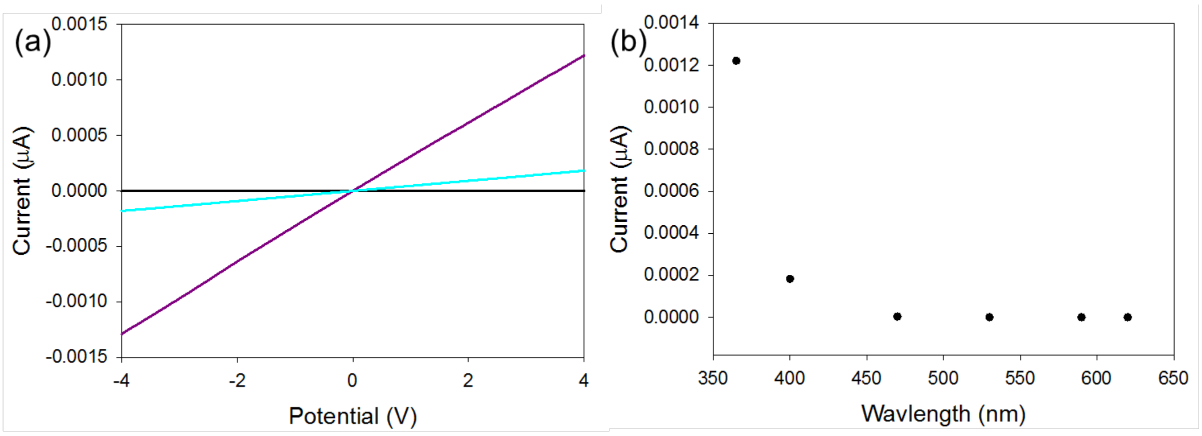

Figure S20. Photoresponse using different wavelengths of light for dried solution of **PBI-W**. (a) I-V curves in the dark (black line), after 365 nm (purple line), 400 nm (light blue line), 450 nm (blue line), 528 nm (green line), 590 nm (yellow line) and 620 nm (red line). (b) Scatter graph showing the wavelength preference of the dried solution of **PBI-W**.

Table S13. Table showing the absolute current generated from the dried films at 4 V under illumination of 365 nm LED for half an hour.

	PBI-A	PBI-F	PBI-H	PBI-L	PBI-S	PBI-V	PBI-W	PBI-Y
**Current at 4 V (μA)**	1.47	3.7 x10^{-6}	0.011	2.4 x10^{-4}	3.9	2.0	1.0 x10^{-4}	1.2 x10^{-3}

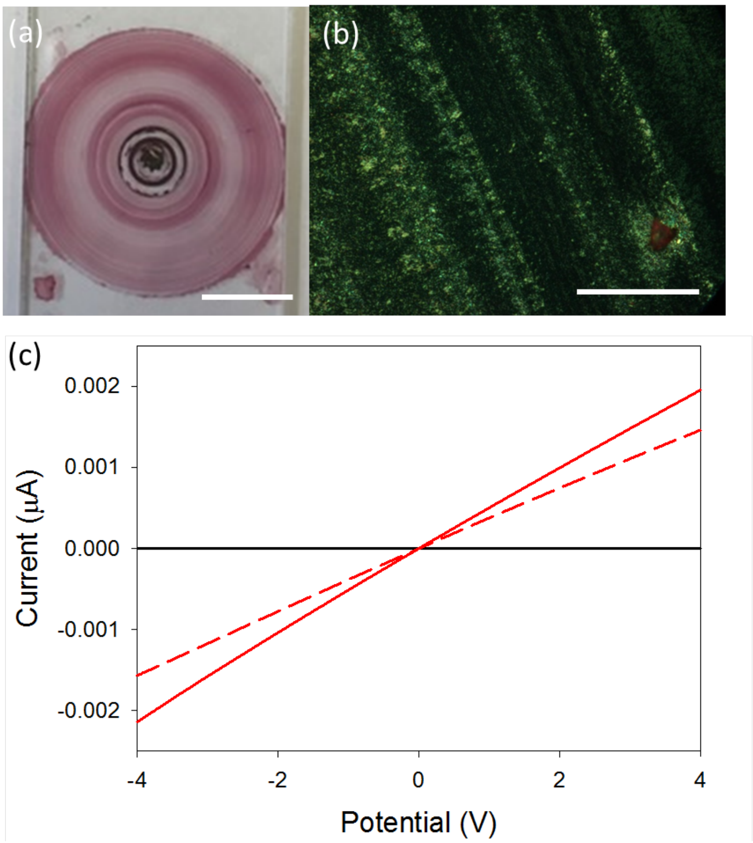

Figure S21. Shear alignment of **PBI-A** solution. (a) photograph of film after shear alignment, scale bar represents 1 cm. (b) Cross-polarised light micrscope image, scale bar respresents 50 μm. (c) I-V curve of the aligned film in

the dark (black data), against the alignment after irradiation with 365 nm (dashed red data) and with the alignment after irradation with 365 nm (solid red data).

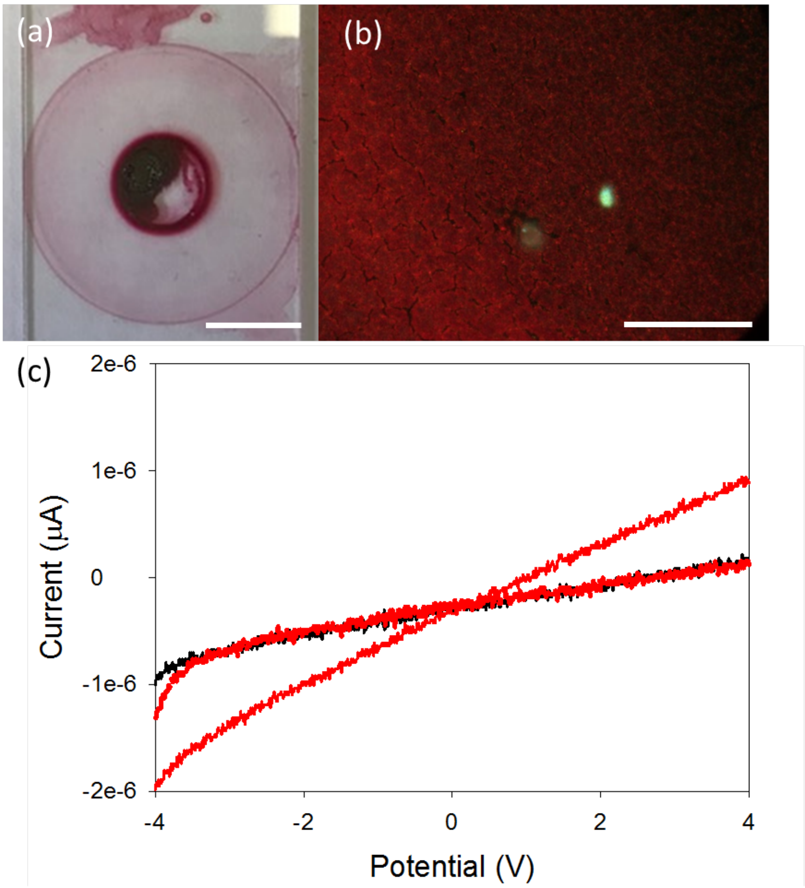

Figure S22. Shear alignment of **PBI-F** solution. (a) photograph of film after shear alignment, scale bar represents 1 cm. (b) Cross-polarised light micrscope image, scale bar respresents 50 µm. (c) I-V curve of the aligned film in the dark (black data), against the alignment after irradiation with 365 nm (dashed red data) and with the alignment after irradation with 365 nm (solid red data).

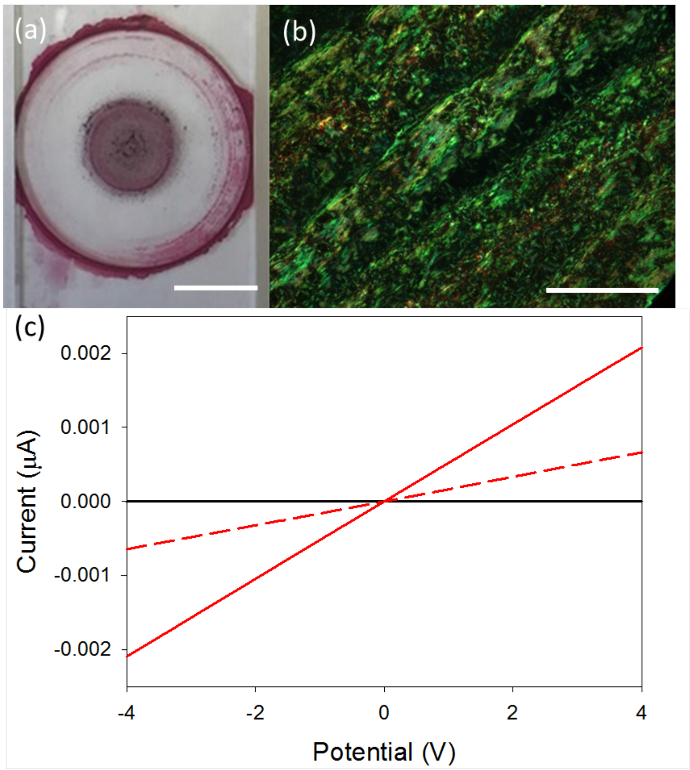

Figure S23. Shear alignment of **PBI-H** solution. (a) photograph of film after shear alignment, scale bar represents 1 cm. (b) Cross-polarised light micrscope image, scale bar respresents 50 μm. (c) I-V curve of the aligned film in the dark (black data), against the alignment after irradiation with 365 nm (dashed red data) and with the alignment after irradation with 365 nm (solid red data).

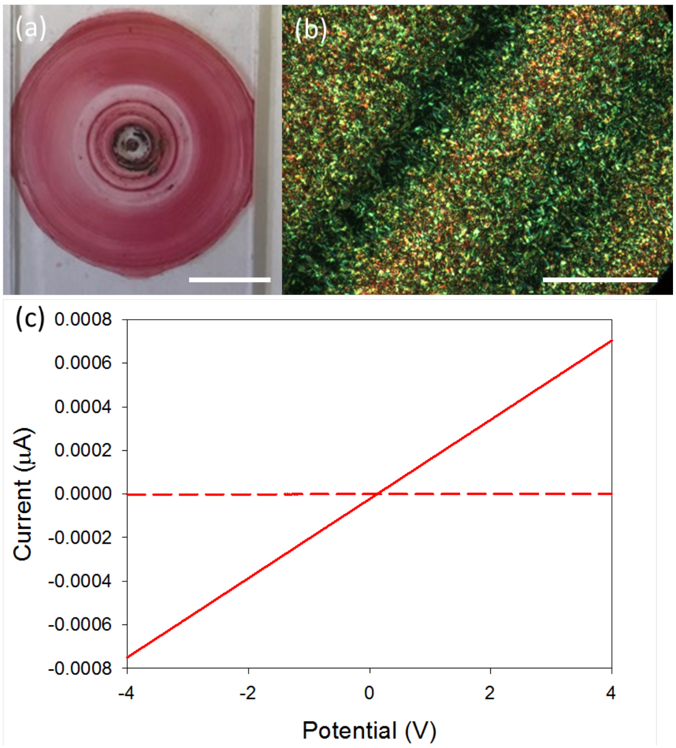

Figure S24. Shear alignment of **PBI-L** solution. (a) photograph of film after shear alignment, scale bar represents 1 cm. (b) Cross-polarised light micrscope image, scale bar respresents 50 μm. (c) I-V curve of the aligned film in the dark (black data), against the alignment after irradiation with 365 nm (dashed red data) and with the alignment after irradation with 365 nm (solid red data).

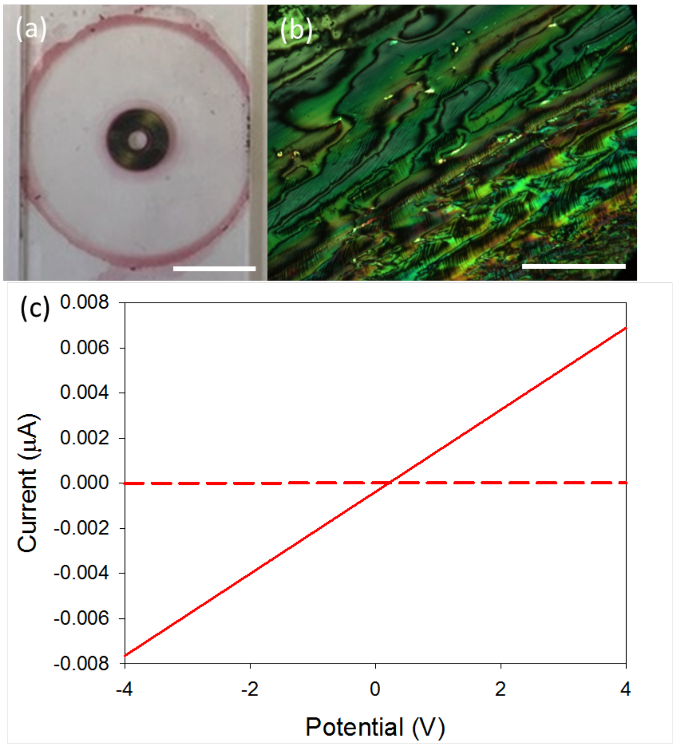

Figure S25. Shear alignment of **PBI-S** solution. (a) photograph of film after shear alignment, scale bar represents 1 cm. (b) Cross-polarised light micrscope image, scale bar respresents 50 µm. (c) I-V curve of the aligned film in the dark (black data), against the alignment after irradiation with 365 nm (dashed red data) and with the alignment after irradation with 365 nm (solid red data).

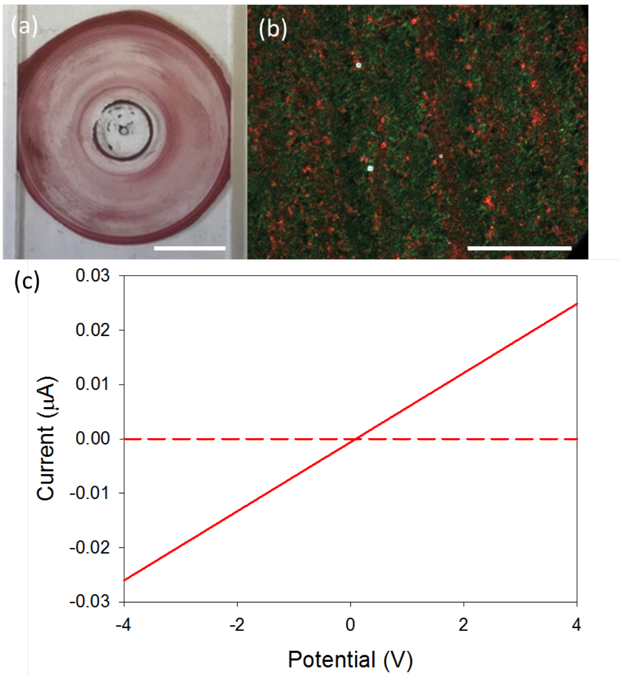

Figure S26. Shear alignment of **PBI-V** solution. (a) photograph of film after shear alignment, scale bar represents 1 cm. (b) Cross-polarised light micrscope image, scale bar respresents 50 µm. (c) I-V curve of the aligned film in the dark (black data), against the alignment after irradiation with 365 nm (dashed red data) and with the alignment after irradation with 365 nm (solid red data).

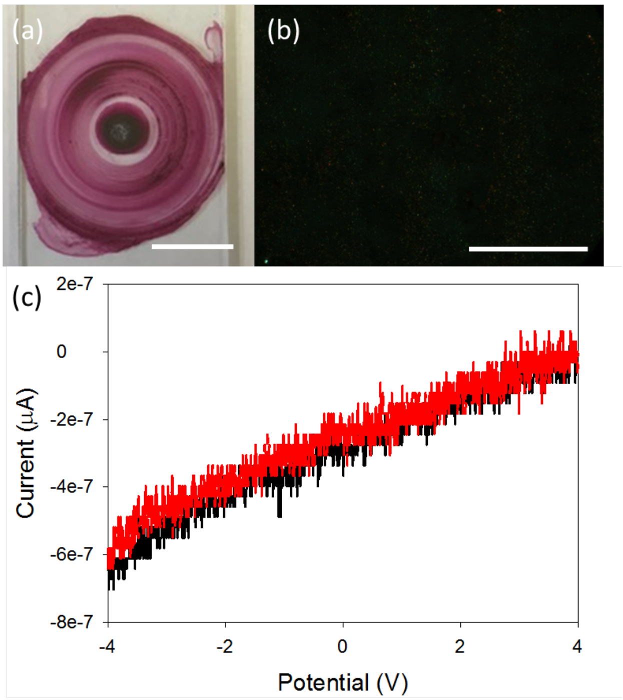

Figure S27. Shear alignment of **PBI-W** solution. (a) photograph of film after shear alignment, scale bar represents 1 cm. (b) Cross-polarised light micrscope image, scale bar respresents 50 µm. (c) I-V curve of the aligned film in the dark (black data), against the alignment after irradiation with 365 nm (dashed red data) and with the alignment after irradation with 365 nm (solid red data).

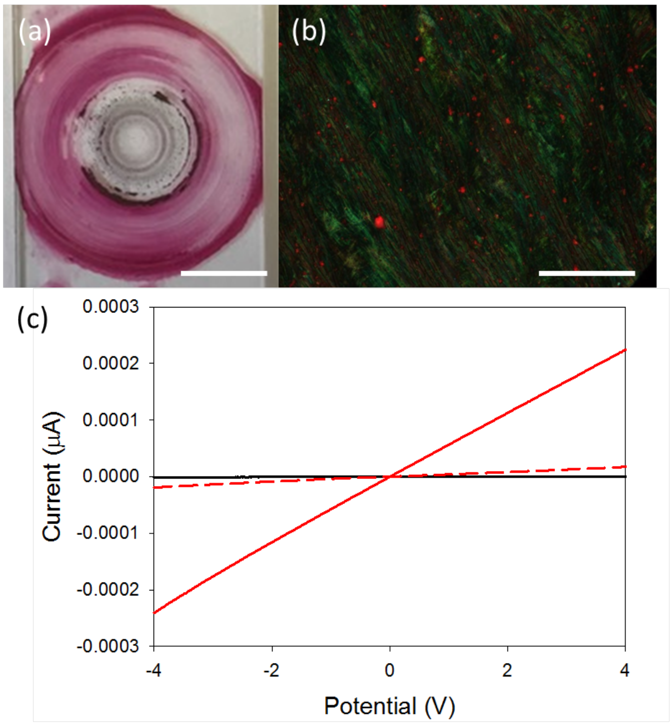

Figure S28. Shear alignment of **PBI-Y** solution. (a) photograph of film after shear alignment, scale bar represents 1 cm. (b) Cross-polarised light micrscope image, scale bar respresents 50 µm. (c) I-V curve of the aligned film in the dark (black data), against the alignment after irradiation with 365 nm (dashed red data) and with the alignment after irradation with 365 nm (solid red data).

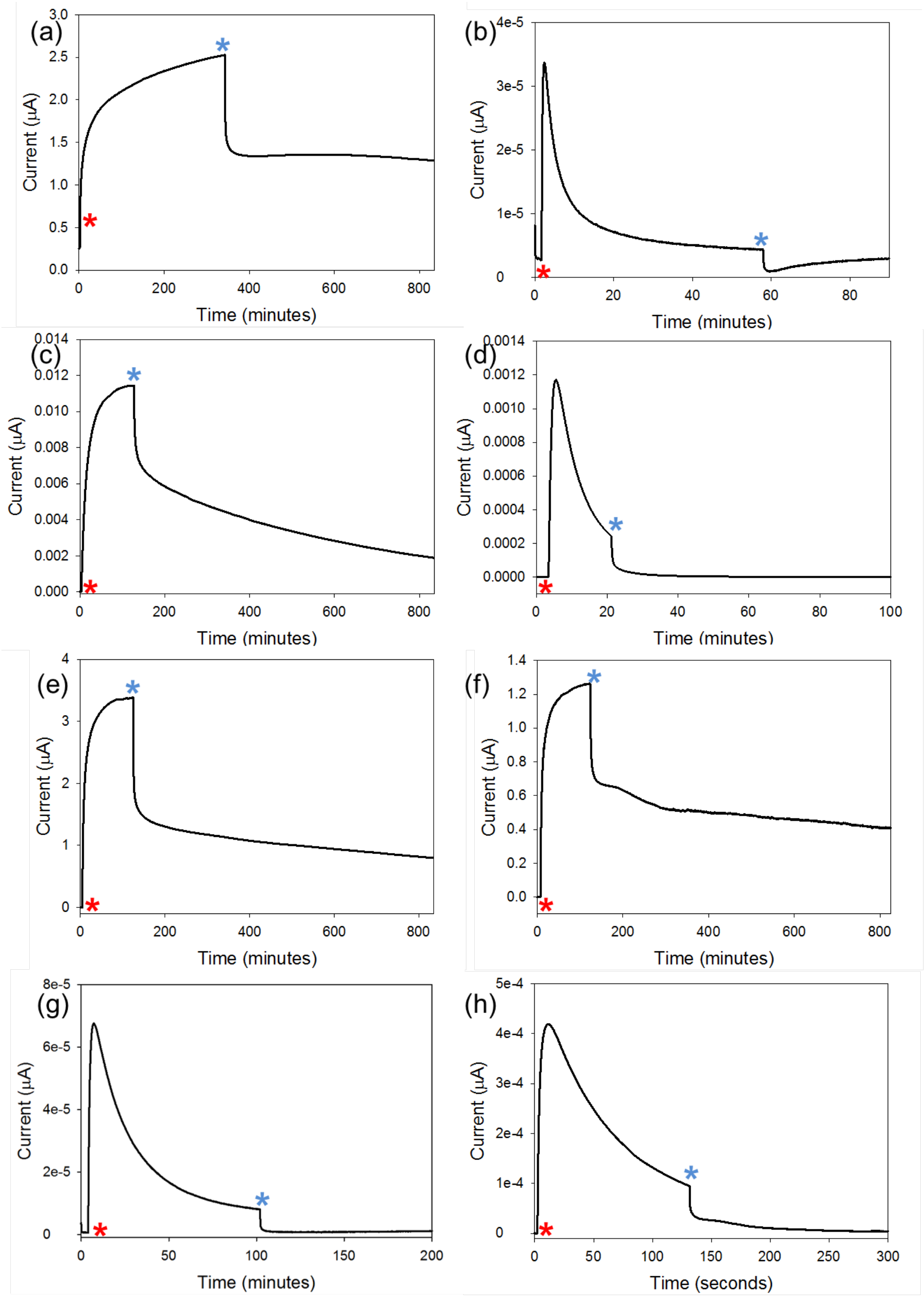

Figure S29. Ampermetric detection at 4 V of dried solution. The red star is when the 365 nm LED is switch on and the blue star when it was switched off. (a) **PBI-A**, (b) **PBI-F**, (c) **PBI-H**, (d) **PBI-L**, (e) **PBI-S**, (f) **PBI-V**, (g) **PBI-W** and (h) **PBI-Y**. The stability of the conductivity of the films was investigated by monitoring the evolution of the photocurrent while illuminating the films for a period before turning the lamp of again. The three samples that showed the presence of the dianion exhibited a longer grow in of the response, followed by a long decay of the current after the light was switched off. The current for the more responsive samples (**PBI-A**, **PBI-H**, **PBI-S**, and **PBI-V**, which have a similar UV-vis absorption spectrum to each other in solution) stabilized after illumination for 100 minutes. This current persisted longer than 10 hours after the light was switched off. However, with the less responsive samples, on irradiation, the current increased and then decreased again before stabilising after around 30 minutes. On turning off the light, the decay of the current then took a maximum of 100 minutes to decay to the dark current. **PBI-F**, **PBI-L**, **PBI-W**, and **PBI-Y** show this effect; these also have similar UV-vis absorption spectra (see main paper). This behavior could be repeated on the same film once the decay to the dark current had been reached, showing that the initial decrease under illumination is not due to film degradation.

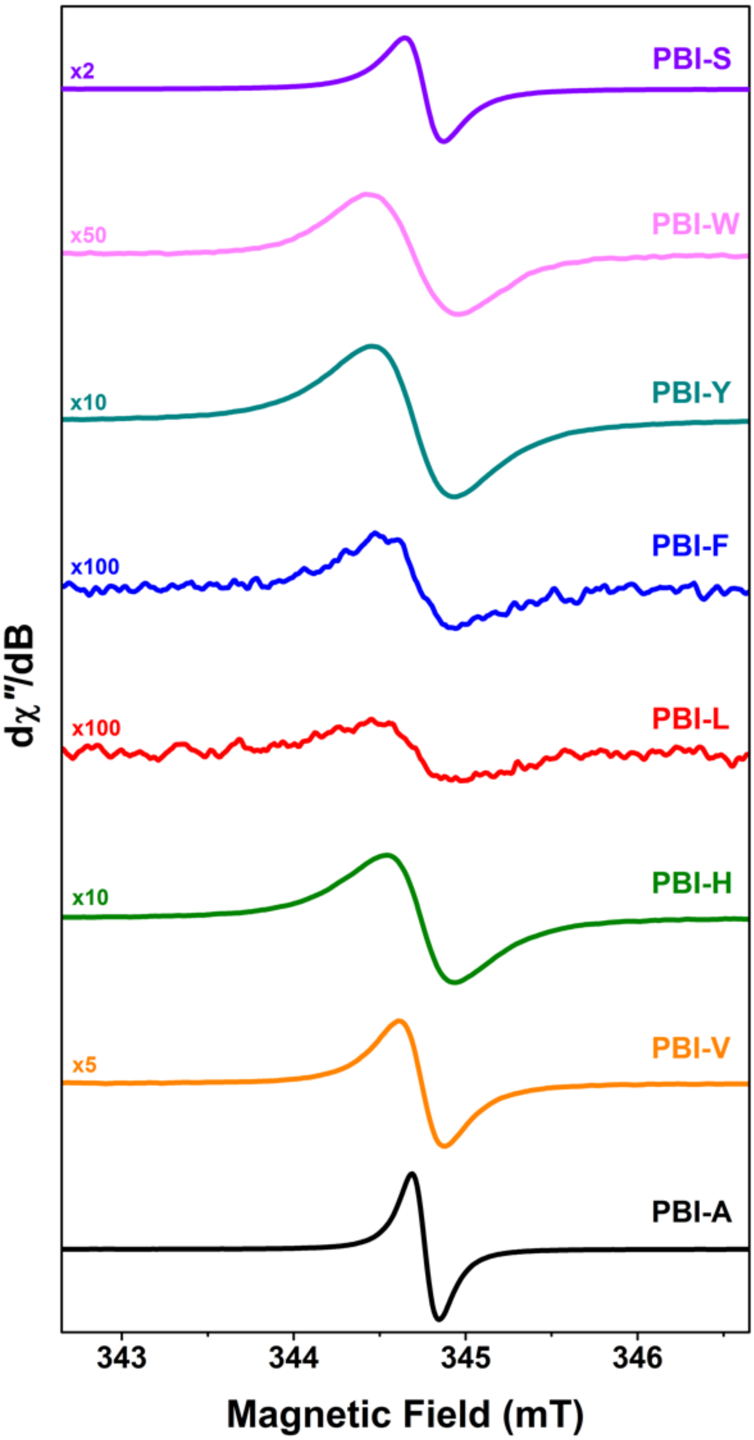

Figure S30. Comparison of the normalized X-band EPR spectra of the PBIs films at ambient temperature after achieving maximum signal intensity post irradiation with 365 nm (experimental conditions: frequency, 9.67 GHz; power, 0.63 mW; modulation, 0.05 mT).

References

[1] a) P. Chal, A. Shit, A. K. Nandi, *Journal of Materials Chemistry A* **2016**, *4*, 16108-16118; b) M. J. Farooqi, M. A. Penick, J. Burch, G. R. Negrete, L. Brancaleon, *Spectrochimica Acta Part A: Molecular and Biomolecular Spectroscopy* **2016**, *153*, 124-131; c) W. Kim, B.-G. Ryu, S. Kim, S.-W. Heo, D. Kim, J. Kim, H. Jo, J.-H. Kwon,

J.-W. Yang, *Korean Journal of Chemical Engineering* **2014**, *31*, 381-385; d) Y. Ma, F. Zhang, J. Zhang, T. Jiang, X. Li, J. Wu, H. Ren, *Luminescence* **2016**, *31*, 102-107; e) G. Paramaguru, N. Nagarajan, R. Renganathan, *Journal of Molecular Structure* **2013**, *1038*, 235-241; f) C. A. Potter, E.-L. Jeong, M. P. Williamson, P. J. F. Henderson, M. K. Phillips-Jones, *FEBS Letters* **2006**, *580*, 3206-3210; g) Y. Xu, S. Leng, C. Xue, R. Sun, J. Pan, J. Ford, S. Jin, *Angewandte Chemie International Edition* **2007**, *46*, 3896-3899; h) S. Roy, D. Kumar Maiti, S. Panigrahi, D. Basak, A. Banerjee, *RSC Advances* **2012**, *2*, 11053-11060; i) E. R. Draper, J. J. Walsh, T. O. McDonald, M. A. Zwijnenburg, P. J. Cameron, A. J. Cowan, D. J. Adams, *Journal of Materials Chemistry C* **2014**, *2*, 5570-5575.

[2] E. R. Draper, O. O. Mykhaylyk, D. J. Adams, *Chemical Communications* **2016**, *52*, 6934-6937.

[3] www.sasview.org.

[4] a) A. A. Isse, A. Gennaro, *The Journal of Physical Chemistry B* **2010**, *114*, 7894-7899; b) S. Trasatti, in *Pure and Applied Chemistry, Vol. 58*, **1986**, p. 955.

[5] a) A. D. Becke, *The Journal of Chemical Physics* **1993**, *98*, 5648-5652; b) P. J. Stephens, F. J. Devlin, C. F. Chabalowski, M. J. Frisch, *The Journal of Physical Chemistry* **1994**, *98*, 11623-11627.

[6] A. Klamt, G. Schuurmann, *Journal of the Chemical Society, Perkin Transactions 2* **1993**, 799-805.

[7] http://www.consultrsr.net/resources/ref/refpotls.htm.

[8] A. Schäfer, H. Horn, R. Ahlrichs, *The Journal of Chemical Physics* **1992**, *97*, 2571-2577.

[9] F. Weigend, R. Ahlrichs, *Physical Chemistry Chemical Physics* **2005**, *7*, 3297-3305.

[10] a) R. Ahlrichs, M. Bär, M. Häser, H. Horn, C. Kölmel, *Chemical Physics Letters* **1989**, *162*, 165-169; b) F. Furche, R. Ahlrichs, C. Hättig, W. Klopper, M. Sierka, F. Weigend, *Wiley Interdisciplinary Reviews: Computational Molecular Science* **2014**, *4*, 91-100.

[11] J. J. Walsh, J. R. Lee, E. R. Draper, S. M. King, F. Jäckel, M. A. Zwijnenburg, D. J. Adams, A. J. Cowan, *The Journal of Physical Chemistry C* **2016**, *120*, 18479-18486.
